# Evolutionary and genomic analysis of the caleosin/peroxygenase (CLO/PXG) gene/protein families in the Viridiplantae

**DOI:** 10.1371/journal.pone.0196669

**Published:** 2018-05-17

**Authors:** Farzana Rahman, Mehedi Hassan, Rozana Rosli, Ibrahem Almousally, Abdulsamie Hanano, Denis J. Murphy

**Affiliations:** 1 Genomics and Computational Biology Research Group, University of South Wales, Pontypridd, United Kingdom; 2 Advanced Biotechnology and Breeding Centre, Malaysian Palm Oil Board, Kuala Lumpur, Malaysia; 3 Department of Molecular Biology and Biotechnology, Atomic Energy Commission of Syria, Damascus, Syria; Saint Mary's University, CANADA

## Abstract

Bioinformatics analyses of caleosin/peroxygenases (*CLO/PXG*) demonstrated that these genes are present in the vast majority of Viridiplantae taxa for which sequence data are available. Functionally active CLO/PXG proteins with roles in abiotic stress tolerance and lipid droplet storage are present in some Trebouxiophycean and Chlorophycean green algae but are absent from the small number of sequenced Prasinophyceaen genomes. *CLO/PXG*-like genes are expressed during dehydration stress in Charophyte algae, a sister clade of the land plants (Embryophyta). *CLO/PXG*-like sequences are also present in all of the >300 sequenced Embryophyte genomes, where some species contain as many as 10–12 genes that have arisen via selective gene duplication. Angiosperm genomes harbour at least one copy each of two distinct CLO/PX isoforms, termed H (high) and L (low), where H-forms contain an additional C-terminal motif of about 30–50 residues that is absent from L-forms. In contrast, species in other Viridiplantae taxa, including green algae, non-vascular plants, ferns and gymnosperms, contain only one (or occasionally both) of these isoforms per genome. Transcriptome and biochemical data show that *CLO/PXG*-like genes have complex patterns of developmental and tissue-specific expression. CLO/PXG proteins can associate with cytosolic lipid droplets and/or bilayer membranes. Many of the analysed isoforms also have peroxygenase activity and are involved in oxylipin metabolism. The distribution of *CLO/PXG*-like genes is consistent with an origin >1 billion years ago in at least two of the earliest diverging groups of the Viridiplantae, namely the Chlorophyta and the Streptophyta, after the Viridiplantae had already diverged from other Archaeplastidal groups such as the Rhodophyta and Glaucophyta. While algal CLO/PXGs have roles in lipid packaging and stress responses, the Embryophyte proteins have a much wider spectrum of roles and may have been instrumental in the colonisation of terrestrial habitats and the subsequent diversification as the major land flora.

## Introduction

Comparative genomic and functional analyses of individual gene families can shed considerable light on the process of plant evolution and on the physiological role(s) of particular groups of proteins[1, 2]. In this study we have analysed a well-conserved gene family, which is normally annotated in databases as ‘caleosin’ and/or ‘peroxygenase’, with the aim of tracing the evolution, expression patterns and functional roles of the encoded proteins in plants. In terms of their functional description, the caleosin/peroxygenases (CLO/PXG) are members of the EC:1.11.2.3 class of oxidoreductases, Pfam reference PF05042. However, the CLO/PXGs also have much broader biological functions and are present in the majority of Viridiplantae taxa for which sequence and/or biochemical data are currently available [3]. The Viridiplantae, which include all of the land plants and green algae are divided into two groups, namely the Streptophyta (Charophyta + Embryophyta) and the Chlorophyta (a highly diverse group of green algae including the Trebouxiophyceae, Chlorophyceae and Prasinophyceae)[4–6]. While CLO/PXGs appear to be ubiquitous in all land plant (Embryophyta) genomes, they are present in some, but by no means all, algal taxa within the Charophyta and Chlorophyta[7]. CLO/PXGs are also found in many fungal taxa but are absent from other the major Opisthokont clades, including animals [8, 9].

The proteins encoded by *CLO/PXG* genes are relatively small (typically 25–30 kDa) and contain a highly conserved single calcium-binding EF hand motif, a lipid-binding domain and two invariant heme-coordinating histidine residues [2, 7, 9–11]. Additionally, there is a region containing several predicted kinase sites proximal to the C terminus [1, 2, 12–14]. These features make up the canonical motifs that are used to classify CLO/PXG proteins. We and others have previously shown that some CLO/PXG isoforms from both plants and fungi can bind to a variety of cellular bilayer membranes, including ER and plasmalemma, via a single transmembrane domain [7, 8, 15, 16]. It has also been shown that other CLO/PXG isoforms bind to the phospholipid monolayer membrane that surrounds intracellular lipid droplets (LDs), possibly via a conserved proline-rich motif [17–20]. It is possible that some CLO/PXG isoforms can bind both to bilayer membranes and LDs, as has been demonstrated with other lipid-binding proteins [21–24]. Experimental studies in several labs have confirmed that CLO/PXGs from both plants and fungi can act as calcium-binding proteins that have specific types of lipid peroxygenase (PXG) activities that require the presence of the heme groups coordinated by two invariant histidine residues [9, 10, 25–28]. This lipid peroxygenase activity is commonly associated with epoxy fatty acid biosynthesis as part of overall oxylipin metabolism in plants [25, 29, 30] as well as a broader series of epoxidation, hydroxylation and aromatization activities on substrates including terpenes and acyl derivatives [31]. In view of their multifunctional roles and database annotations as both ‘caleosins’ and ‘peroxygenases’, we will refer to these genes/proteins as *CLO/PXG* and CLO/PXG respectively.

To date, only a relatively small fraction of the many hundreds of plant and fungal genes that are currently annotated as ‘caleosin’ and/or ‘peroxygenase’ in public databases, such as NCBI or Ensembl Plant, have been shown to encode proteins with experimentally proven PXG activity. Moreover, our detailed manual curation of these annotated genes and their derived protein sequences has shown that in some cases these putative CLO/PXG-like sequences lack critical residues known to be involved in key biological functions of the proteins, such as calcium binding, heme coordination or membrane attachment. One of the unusual features of CLO/PXG proteins is that, in addition to often being active enzymes, they can also have important structural roles in cytosolic LDs where they are the second most highly abundant components (after oleosins) in the LD proteome [22, 24]. Indeed, CLO/PXGs have been shown to play important structural roles in facilitating the assembly, stabilisation, storage and turnover of LDs in a range of plant tissues from leaves and seeds to pollen grains and even in individual algal cells [8, 9, 18, 20, 32].

Experimental studies and transcriptional data have implicated CLO/PXGs in a wide range of physiological functions in plants, including a host of processes in vegetative tissues of plants and algae. These physiological processes include drought and osmotic stress responses [33–37], pathogen responses [33, 38], toxin sequestration [39], stomatal regulation, water transpiration, seed germination and G protein signalling [40], nitrogen deprivation [14, 20, 41–43] and adaptation to darkness [44]. In reproductive tissues, such as in seeds and pollen grains, CLO/PXGs have been shown to have roles in lipid packaging and post germinative LD mobilization[16, 24, 45–48].

The purpose of this study was to characterise the *CLO/PXG* gene superfamily in terms of its occurrence in the Viridiplantae, its possible evolutionary origins and to investigate how this might shed light on the biological roles of the encoded proteins. Of particular interest was whether separate CLO/PXG isoforms are involved in the mainly LD-associated structural functions as compared the peroxygenase functions of the proteins, which tend to be associated with bilayer membranes rather than LDs. To achieve this we performed a comprehensive bioinformatic analysis of the >1300 CLO/PXG-related sequences from Viridiplantae species that are currently lodged in public databases. The primary aim of this analysis was to establish a robust phylogeny and to explore the possible evolution of plant CLO/PXGs over the past >1 billion years. Alongside this analysis, we analysed the transcriptional profiles of *CLO/PXGs* in two unpublished monocot (palm) species in addition to analysing profiles from other species that were obtained from public databases.

## Methods

### Transcriptional analyses

Date palm (*Phoenix dactylifera* L.) seeds were collected from fruits of the Sukary cultivar, imported from Kingdom of Saudi Arabia. Seeds were isolated, washed, air-dried and stored in plastic bags at room temperature until required. Seeds were then germinated *in vitro* in a current of running water for two weeks before planting in culture boxes placed in an incubator at 30 ± 2°C and humidified daily and seedlings were obtained 15 days after sowing. Seedlings with a radicle length of 0.5 or 2 or 4.5 cm were referred as stage I, II and III, respectively. For stress experiments, 2,3,7,8-tetrachlorodibenzo-p-dioxin (2,3,7,8-TCDD dissolved in toluene at 10 μg mL^-1^, purity 99%) was purchased from Supelco Inc., USA. TCDD was placed in a 10 mL capped glass tube and evaporated to dryness under nitrogen. For health and environmental safety reasons, residual TCDD was re-dissolved in a minimum volume (100 μL) of dimethyl sulfoxide (DMSO), and 5 mL of aqueous solutions of TCDD were prepared in deionized and distilled water to obtain initial concentrations of 0, 10, 50 and 100 ng L^-1^ TCDD. For treatment of seeds with TCDD, seeds were germinated as described above and humidified daily with the prepared solutions of TCDD at various concentrations. Seedlings at stages 0, I, II and III were taken for further analysis. For drought stress, seedlings were pre-treated with water for 2, 4 and 6 days under the culture conditions described above. Osmotic stress was achieved by irrigating seedlings with water at concentrations of 150 and 300 mg L^-1^ NaCl at each development stage.

Changes in relative transcriptional abundance of genes encoding LD-associated proteins in response to TCDD exposure were analyzed by reverse-transcription quantitative PCR (RT-qPCR). Briefly, frozen fine powder (1 g) samples from whole seedlings at stages 0, I, II and III were used to extract total RNA using an RNeasy kit (Qiagen) according to the manufacturer’s instructions. The quality of extracted RNAs was checked on agarose gels and concentrations measured by a Nanodrop device. Remaining traces of genomic DNA were digested by DNase I and the lack of trace genomic DNA in total RNA was confirmed by a control PCR using total RNA as the template. Aliquots of 1 μg total RNA were used for first-strand cDNA synthesis according to Hanano et al (2014). Real-time and qPCRs were performed as described by Hanano et al (2014) and primers are listed in S1 Table. The relative expression of target genes was normalized using two reference genes *Act-1* and *Tub-β* [49]. Each measurement was performed in triplicate together with a dilution series of the reference gene. PCR efficiencies were between 95% and 105% (data not shown) and the average of *C*_T_ was taken. The relative quantification RQ of target genes was calculated directly using software from the qPCR system. Sequences of amplified regions were confirmed by sequencing on an ABI 310 Genetic Analyzer using a Big Dye Terminator kit (Applied Biosystems).

Oil palm transcriptome analysis was carried out according to [50]. The oil palm genome P5-build was used to read map the RNA seq data from Roche 454 reads (a full dataset is available from NCBI BioProject PRJNA201497). Briefly, reads from Roche/454-derived libraries were assembled into isotigs, which were blasted onto *Arabidopsis thaliana* gene models with a threshold of *E*-value < 10^−5^. The best-hit *A*. *thaliana* gene model was assigned to the homologue of the query isotig. To estimate expression levels of genes in mesocarp and kernel tissues, Illumina HiSeq 2000 reads from each library were mapped to assembled isotigs from all *Elaeis guineensis* reads by using the Burrows–Wheeler Aligner. Gene group expression levels were calculated as the number of mapped reads on each isotig divided by the total number of isotigs, multiplied by 100,000, and scaled by the number of genes in each gene group. Both copy number and read coverage were the mean of measures from two biological replicates. Data were analysed as described above for Roche/454 data, except that expression levels were calculated as transcripts per million tags. Identification of expression caleosin in oil palm was done using open source Tuxedo suite software [51].

### Bioinformatics procedures

The bioinformatics procedures used in this study are summarised in the workflow depicted in S1 Fig. For data collection, a list of *CLO/PXG*–like genes was identified using one model species (*A*. *thaliana)* and three economic crops (*P*. *dactylifera*, *E*. *guineensis*, *Musa acuminate)*, which were then used as the source of the *CLO/PXG* reference sequences in this study. Using reference genomes and proteomes from public databases (i.e. NCBI Entrez and Pubmed), we constructed a dataset of well-annotated full-length *CLO/PXG* genes. We then selected 34 species from the Viridiplantae based on the quality of their sequenced genomes, their economic and conservation importance, and their evolutionary significance. The selected species included representatives from all available major Viridiplantae groups, such as the algal Charophyte, Chlorophyceae and Trebouxiophyceae clades, plus the major Embryophyte (land plant) taxa including bryophytes, ferns, gymnosperms, basal angiosperms and the major extant monocot and dicot groups. Although the vast majority of currently available sequence data comes from the monocot and dicot groups, and particularly from crop species of economic importance, we are confident that there is sufficient genomic data from the sequenced non-angiosperm groups to enable robust conclusions to be drawn about the evolution of this gene family in the Viridiplantae.

### Finding and assessing candidate CLO/PXG sequences from representative genomes

Using sequences from the 34 reference species shown in S2 Table, we performed local alignment searches within each species using blastp from the BLAST+ toolset [52]. At this stage the total number of candidate sequences was >500. These sequences were analysed using InterProScan (http://www.ebi.ac.uk/interpro/) to confirm the presence of the calcium binding and EF hand domains. *CLO/PXG* sequences were also visually inspected using Geneious version 10.0.9 (http://www.geneious.com, and CLC Genomics Workbench 10.0.3 (https://www.qiagenbioinformatics.com/) to confirm the presence of the full range of canonical *CLO/PXG* domains [53, 54]. After confirming the presence of major caleosin domains, we derived a list of candidate caleosin genes and assigned names to these sequences, which are shown in S2 Table with corresponding species names and clade/groups. We then performed a set of experiments using published and peer-reviewed toolsets on these candidate sequences. The experiments included motif discovery, physical and chemical property analysis, domain sequence conservation analysis and consensus study and evolutionary pattern studies. This enabled us to narrow down the dataset to 131 *CLO/PXG*-like sequences. A list of physical and chemical properties of CLO/PXG proteins (molecular weight (MW), isoelectric point (pI), amino acid length) is shown in S2 Table. The physical and chemical properties of CLO/PXG proteins were computed using ExPASY (http://www.expasy.org/protscale/) [55, 56]. For further detailed analysis we selected 67 of the 131 sequences that presented themselves as strong candidates to belong to the CLO/PXG family (S2 Table where sequences marked in green are the 67 selected CLO/PXGs). A list of the 34 species with their corresponding CLO name, Taxon ID and number of CLO/PXG isoforms per species is given in S3 Table.

### Motif, transmembrane (TM) domain, secondary structure prediction and intron-exon analysis

**Fig 1:**
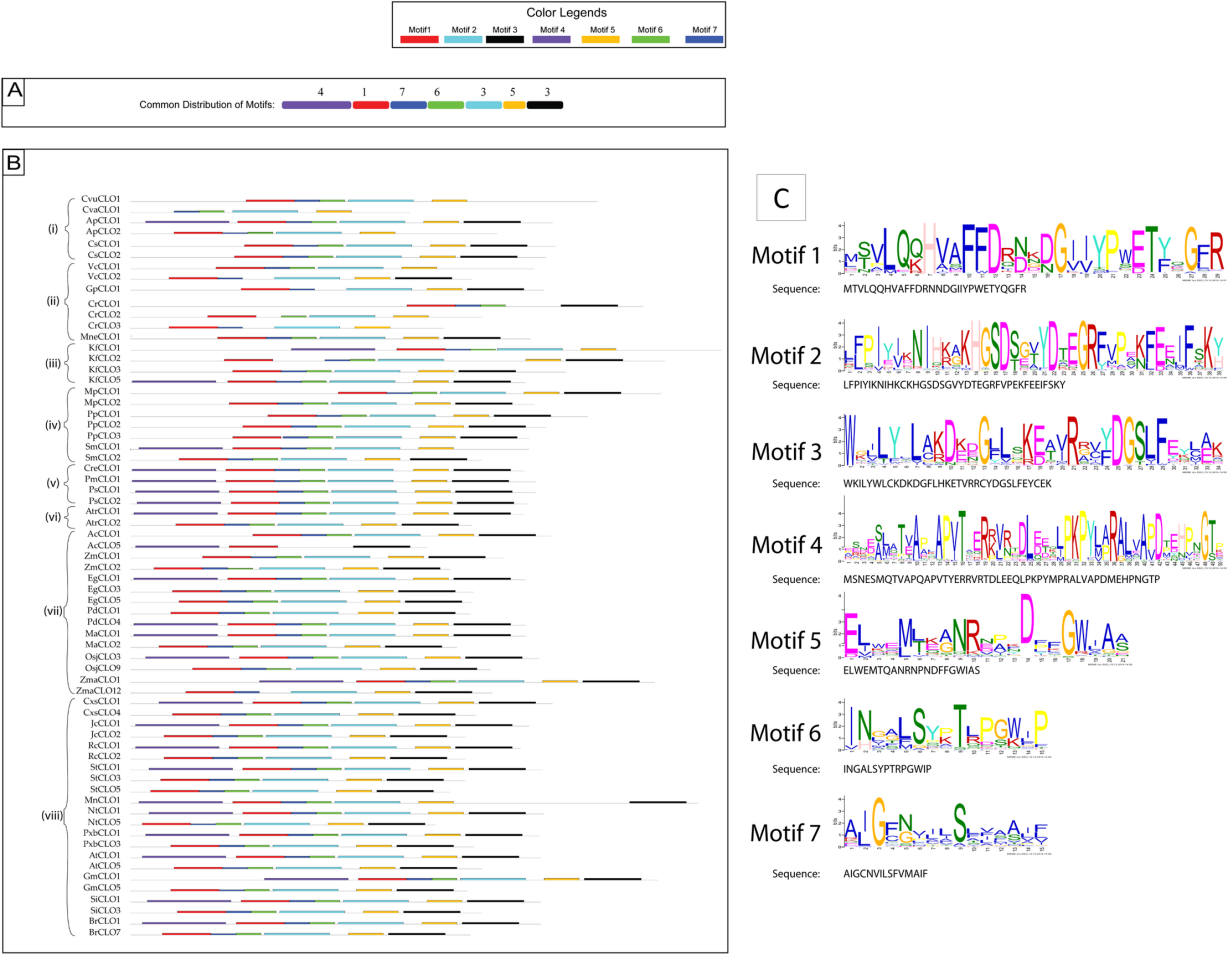
Motif analysis. (A) Consensus distribution of motifs in all Viridiplantae sequences. (B) Distribution of motifs across 67 representative Viridiplantae CLO/PXG proteins grouped in eight taxonomic clades as follows. (i) Trebouxiophyceae, (ii) Chlorophyceae, (iii) Charophyta, (iv) Non-seed plants, (v) Gymnosperms, (vi) basal Angiosperms, (vii) Monocots and (viii) Dicots.(C) Sequences of the 7 major motifs found in Viridiplantae CLO/PXG proteins.

Motif analysis was performed on the 67 sequences from 34 species to identify conserved domains that might elucidate the biological activities of these multifunctional proteins. The Multiple En for Motif Elicitation (MEME) software package was utilised to discover and analyse motifs across sequences [57, 58]. Protein datasets were used to analyse motifs for each sequence. Sequences were analysed using the discriminative mode and a window size of 15–50, which enabled identification of seven distinctive motifs. The list of motif logos and the motif distribution patterns across species sequences are shown in Fig 1. Transmembrane (TM) domain and secondary structure predictions were performed using the Geneious package and both Geneious and ClustalW aligners were used to align and prepare the sequences for display. The following parameters were used to align protein sequences: a) Cost matrix BLOSUM 45, b) Gap open penalty 12, c) Gap extension penalty 3, and d) refinement iteration of 2 and the resulting data are shown in S2 Fig. The location (start and end point) of each transmembrane domain is shown in S4 Table and S2 Fig. The lengths of the TMs are consistent at 21 residues. The secondary structure predictions are shown in S3 Fig. The Scipio program version 1.4 was used to identify intron-exon [59]. A summary graph of overall intron/exon numbers identified using the Scipio program in each sequence is shown in S4 Fig while S5 Table shows the locations of intron/exons in the full list of the 67 analysed genes.

### MSA (multiple sequence alignment) and phylogenetic analyses

**Fig 2:**
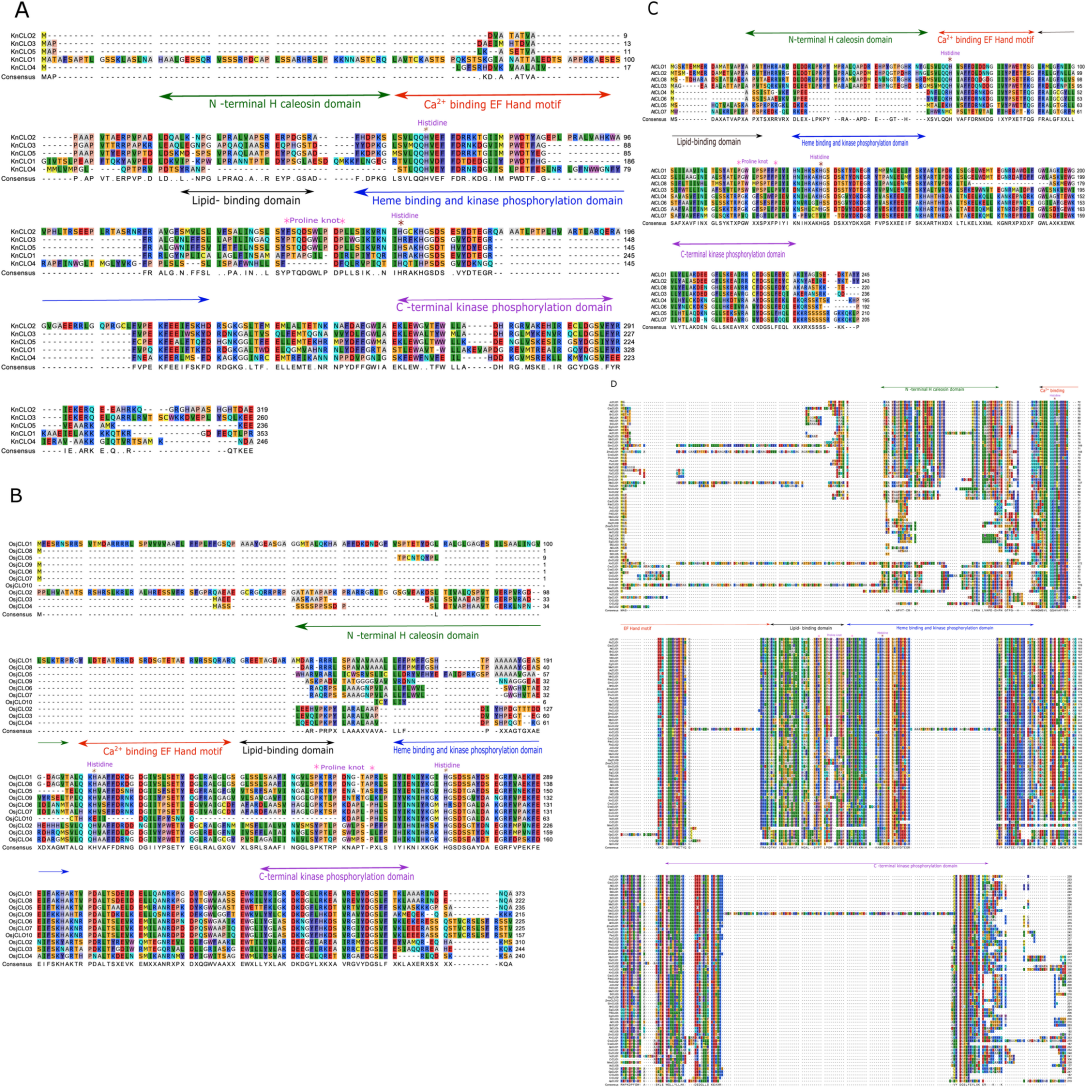
Sequence alignments of CLO/PXG protein families from three representative Charophyte species plus 67 sequences from 34 species across the Viridiplantae. (A) *Klebsormidium nitens* alignment. (B) *Oryza sativa* alignment. (C) *Arabidopsis thaliana* alignment. (D) 67 protein sequences alignment from 34 Viridiplantae species. The five major structural domains are shown respectively as the N-terminal H-caleosin, Ca 2^+^ binding EF Hand, Lipid-binding, Heme binding and kinase phosphorylation and C-terminal kinase phosphorylation domains. The proline knot region and two conserved Histidines are also shown.

**Fig 3:**
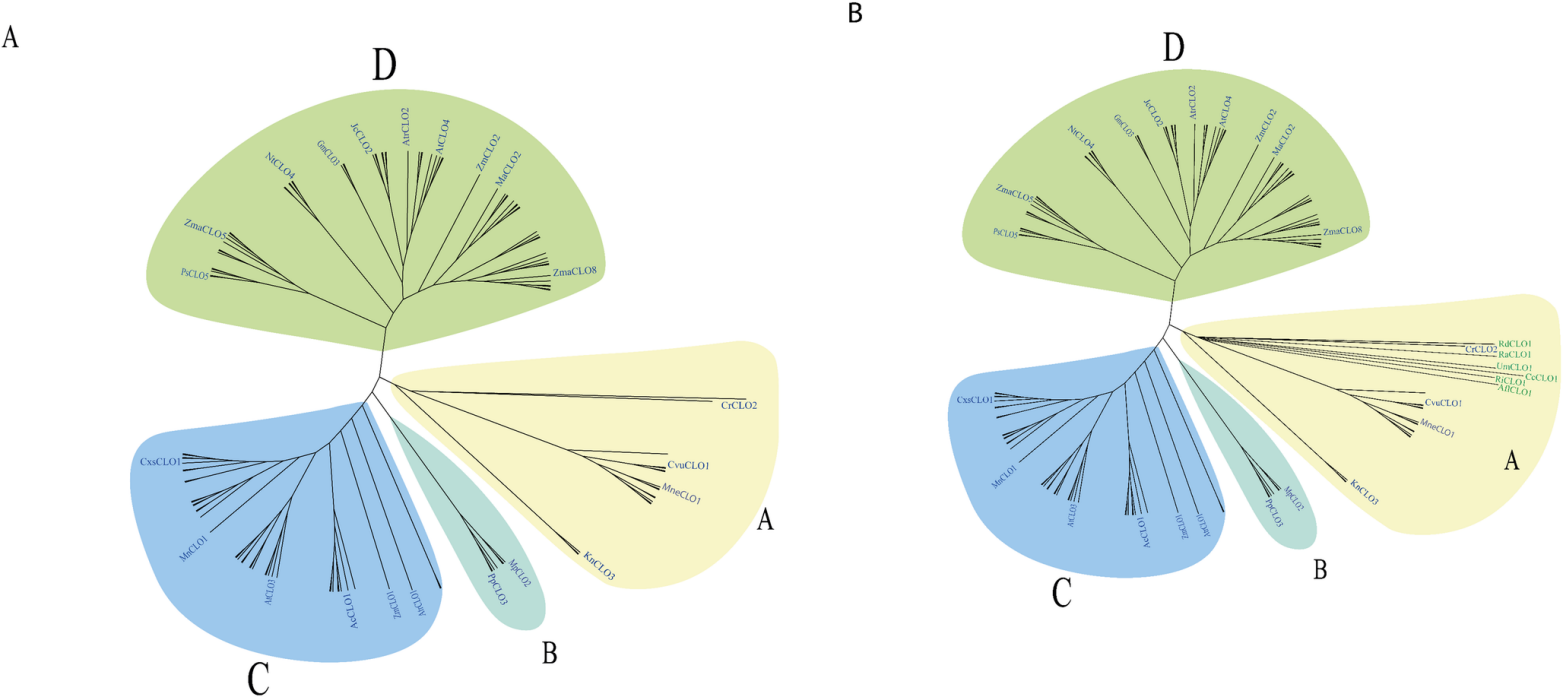
Phylogenetic analysis of 67 CLO/PXG sequences. (A) 67 CLO/PXG sequences from 34 species across the Viridiplantae. (B) 67 CLO/PXG sequences from 34 species across the Viridiplantae plus six selected fungal species.

Multiple sequence alignment and domain analyses were performed using ClustalOmega software, version 1.2.2, using the default parameters [60, 61]. The alignments were inspected using the CLC Genomics Workbench 10.0.3 (https://www.qiagenbioinformatics.com/). Complete alignments with RasMol colour codes [62] are shown in Fig 2. The amino acid sequence alignments were used to construct a phylogenetic tree using the ClustalW2 program version 2.1. The tree was generated following Bayesian Inference (BI), Neighbour-joining (NJ), and Unweighted Pair Group Method with Arithmetic Mean (UPGMA) methods. The tree topologies constructed using the three different methods showed complete consistency. The NJ tree was constructed using ClustalW2 program version 2.1 [60]. The constructed tree was inspected using FigTree and is shown in Fig 3 [63]. Phylogenetic analysis shown in Fig 3A was built from CLO/PXG sequences across Viridiplantae while the tree shown in Fig 3B was built with CLO/PXG sequences across Viridiplantae plus a range of representative basal and more advanced Fungal species, namely *Aspergillus flavus*, *Ustilago maydis*, *Rozella allomycis*, *Coprinus cinereus*, *Rhizophagus irregularis*, and *Rhizopus delemar*. Note that the addition of these six fungal sequences did not affect the phylogeny of the plant sequences.

## Results and discussion

### Bioinformatics analyses

As of November 2017 we found >1310 sequence hits from the Viridiplantae that were classified in public databases, such as NCBI, as being members of the ‘caleosin superfamily’. These sequences were mostly annotated in databases as either ‘caleosin’ or ‘peroxygenase’ although some were labelled as ‘hypothetical protein’, and a small number were labelled as ‘ABA-induced protein’ or ‘EF hand protein’. An example of the annotation list for the CLO/PXG enzymes (EC:1.11.2.3) in *A*. *thaliana* can be found at http://www.brenda-enzymes.org/all_enzymes.php?ecno=1.11.2.3&table=Source_Tissue#TAB. It should be noted that although this list is annotated as ‘plant seed peroxygenase’, the source tissue list shows that the relevant *CLO/PXG* genes are expressed throughout the plant and not just in seeds. We found that some of the putative *CLO/PXG* genes in the various public genome databases were present as incomplete or partial sequences with missing and/or corrupted versions of the major canonical CLO/PXG domains known to be essential for protein function. Such sequences, which may be from non-functional pseudogenes, were normally discarded in our analysis although several examples were included to illustrate points of gene expansion as discussed below. A list of the chosen representative 34 species across the Viridiplantae and the *CLO/PXG* sequences used in subsequent analyses in shown in S2 Table.

As shown in Fig 1C, motif analysis using MEME enabled the identification of seven highly conserved CLO/PXG protein regions, which varied in length from 15 to 50 residues, and are found throughout the Viridiplantae. The motifs are numbered 1 to 7 in order of their motif score, which reflects their length and their extent of conservation across all the species analysed. The distribution of these motifs beginning at the N terminus of CLO/PXG proteins was 4-1-7-6-2-5-3 as shown in Fig 1A. In Fig 1B, the selected CLO/PXGs are grouped in eight distinctive taxonomic clades that represent the major Viridiplantae groups. In all cases these highly divergent plant groups show highly conserved organisation of the seven Motifs listed above. However, it should be noted that Motif 4, which is present in H-domain variants, is absent from at least one CLO/PXG sequence from all angiosperm species, i.e. Groups (vi), (vii) and (viii), and it is these Motif 4-lacking proteins that make up the L-isoforms of CLO/PXG [1, 2, 64, 65]. Motif 4 is a 30–50 residue domain present close to the N-terminus and is found in many CLO/PXG sequences throughout the Viridiplantae (and Fungi). This Motif is characteristic of the so-called H-caleosins (where H = high molecular weight), as previously reported from several labs [1, 2, 27, 28, 64–66]. We found that all Angiosperm genomes sequenced to date contain at least one copy each of the L- and H-caleosin sequences and although the evidence is less clear for the other Viridiplantae, it seems that most species also contain both isoforms. One reason for the existence of these two isoforms that are differentiated only by the 30–50 residue N-terminal insertion may be found in their respective pI values. The H-isoforms have low pI values typically below 6 while the L-isoforms have values typically above 8, which indicates that they may function optimally in different subcellular compartments [1].

Motif 1 includes the canonical calcium binding EF hand domain as found in all CLO/PXG sequences in all species. Motifs 7 and 6 contain the proline-rich and lipid binding domains respectively, which are well conserved in the Viridiplantae. Although the MEME software identified Motifs 7 and 6 as separate features, they can probably be regarded functionally as a single well conserved lipid-binding motif due to the presence of the group of about 20 non-polar residues that make up the putative TM or LD-binding domain. Motif 2 includes heme-binding and kinase phosphorylation domains and includes a relatively lengthy 32-residue consensus sequence, NIHKCKHGSDSGVYDTEGRFVPEKFEEIFSKY, which we also found as a very highly conserved domain in most plant and fungal CLO/PXGs. Motif 5 is a shorter and weaker feature that is of unknown function. Motif 3 is a well-conserved C-terminal domain with a characteristic casein kinase phosphorylation box, DGSLFE, as reported elsewhere [12, 40]. However, the full version of Motif 3 includes a larger 27-residue consensus sequence, LYWLCKDKDGFLHKETVRRCYDGSLFE, which is relatively highly conserved across the Viridiplantae.

The transmembrane domain (TM) predictions (S4 Table) and (S2 Fig) show the distribution of TM domains in each CLO/PXG sequence. Note that, since many CLO/PXG isoforms bind to LDs either instead of or in addition to bilayer membranes, these TM domains can be regarded as generalised lipid binding domains and not necessarily only involved in transmembrane functions. A single TM domain was present in each case except for five sequences where there were two TM domains. These five CLO/PXG sequences were ApCLO1 from Trebouxiophyceae, VcCLO1 and GpCLO1 from Chlorophyceae, SmCLO2 from lower plants and EgCLO3 from the monocot group. There were also six CLO/PXG sequences where no predicted TM domain was present, namely CrCLO2 from Chlorophyceae, MpCLO1 from lower plants, PdCLO4 from monocots, StCLO5, NtCLO5 and GmCLO5 from dicots. In all cases these were members of larger CLO/PXG families in each species and, since the predicted proteins had all the other features of CLO/PXGs, these may be non-lipid-binding, soluble isoforms. Gene structures were predicted using the Scipio program version 1.4. Identified intron-exons were inspected using Webscipio program and are presented in S5 Fig [67, 68]. A summary graph of overall intron/exon numbers identified using webscipio program in each sequence is shown in S4 Fig while S5 Table shows the detailed locations of intron/exons in the full list of the 67 analysed genes.

This shows that their gene organisation is relatively divergent in the Viridiplantae as a whole, although the intron/exon structures are relatively conserved within the more recently diverged dicot group of species.

In Fig 2, the protein sequence alignments are shown for CLO/PXG protein families from three representative Charophyte species, namely the alga *Klebsormidium nitens* (Fig 2A) which contains 5 isoforms, the monocot *Oryza sativa* (Fig 2B) with 10 isoforms, and the dicot *A*. *thaliana* (Fig 2C) with 7 isoforms. For a full sequence alignment of all 67 representative CLO/PXG proteins from 34 Viridiplantae species see Fig 2D. All of these alignments show high levels of sequence conservation, especially within the seven key motifs depicted in Fig 1. The three alignments in Fig 2 demonstrate how *CLO/PXG* gene families can be relatively large and the encoded proteins rather diverse, even within a single species. This is consistent with transcriptome and functional evidence that different CLO/PXG proteins are found in different subcellular and tissue locations, are subject to different forms of regulation and also may have different types of enzymatic or structural activities in plants [8, 27, 28, 69, 70]. In the case of the alga *K*. *nitens* (Fig 2A), the major structural domains are conserved in all five isoforms and while there are large insertions in three of the isoforms, these do not affect the integrity of the key canonical domains. For example KNCLO1 has a 100+ residue extension at the N terminal, which has no similarity with any other published sequence, but the remainder of the protein is obviously a member of the CLO/PXG family.

The monocot *O*. *sativa* (japonica rice) genome contains 10 annotated CLO/PXG-like sequences (Fig 2B), not all of which are likely to be functional proteins. In particular, OsjCLO10 lacks the Ca^+^-, heme- and lipid- binding domains and contains a 150-residue insert at the N-terminus. This insert has some similarity with the missing CLO/PXG-like domains and may therefore be due to a transposition of part of the genomic sequence, most likely due to exon shuffling. In contrast, OsjCLO2 contains a 68-residue insert at the N-terminus with no homology to other database sequences but the remainder of the protein includes all the normal CLO/PXG motifs. Apart from these unusual sequences, the remaining 8 rice isoforms show the normal protein architecture although their H-domains are much less conserved compared to those of *A*. *thaliana* as discussed below. Transcriptome data show that 8 of the rice *CLO/PXG*-like genes are expressed in a wide variety of vegetative and reproductive tissues and are modulated by drought, salt and cold stresses [71].

The model dicot species *A*. *thaliana* contains 7 CLO/PXG isoforms (Fig 2C). According to transcriptome data, all of the genes encoding these isoforms are expressed, although *AtCLO7* is only present at very low levels relative to the other 6 genes [1, 65]. As in other Brassicaceae species, it is clear that segmental duplication is the main driver of the accumulation of the large gene family in *A*. *thaliana* [1, 65]. However, a more detailed inspection of the AtCLO7 sequence shows that the protein is not a true caleosin because it lacks critical motifs such as the Ca^+^-binding EF hand and the two heme-binding histidine residues. In comparison with the other six genes, *AtCLO7* is the least expressed and shows little or no response to environmental stimuli [1], which means that it is probably a pseudogene. The other six sequences fall into two well-defined classes, namely the H-isoforms, AtCLO1, 2 & 3 plus AtCLO8, and the L-isoforms, AtCLO4 & 6.

Phylogenetic analyses of a representative 67 CLO/PXG sequences from 34 species across the Viridiplantae are shown in Fig 3A. The sequences separate into four clearly distinct clusters that are labelled as follows: A) Chlorophyte and Streptophyte green algae, B) non-angiosperm Embryophytes; C) H-isoform angiosperms; and D) L-isoform angiosperms. This phylogeny supports the conclusion from the Motif analysis (see above) that H-caleosin isoforms are ancestral to the L-caleosin isoforms. In Fig 3B, the phylogenetic analysis has been extended to include sequences from six selected basal and advanced species that represent all of the major Fungal taxa as well as the 67 Viridiplantae sequences. The fungal sequences used were: *A*. *flavus* (AflCLO1); *U*. *maydis* (UmCLO1); *R*. *allomycis* (RaCLO1); *C*. *cinereus* (CcCLO1); *R*. *irregularis* (RiCLO1); and *R*. *delemar* (RdCLO1). Note that the CLO/PXG sequences for each of these very diverse fungal species that probably diverged from one another >1 billion years ago (Bya) [72, 73] are located within a single branch of the green algal cluster and fall within the same group as the Trebouxiophyte, (shown as the yellow group A). This indicates that the fungal CLO/PXG sequences are more closely related to those of algae than to the land plants, which did not appear until after 500 million years ago (Mya), and the fungal CLO/PXGs may therefore have been derived from Trebouxiophyte algae. The origin, distribution and biological functions of fungal CLO/PXGs will be the subject of a subsequent paper.

### Transcriptional analyses of CLO/PXG in date and oil palm

**Fig 4:**
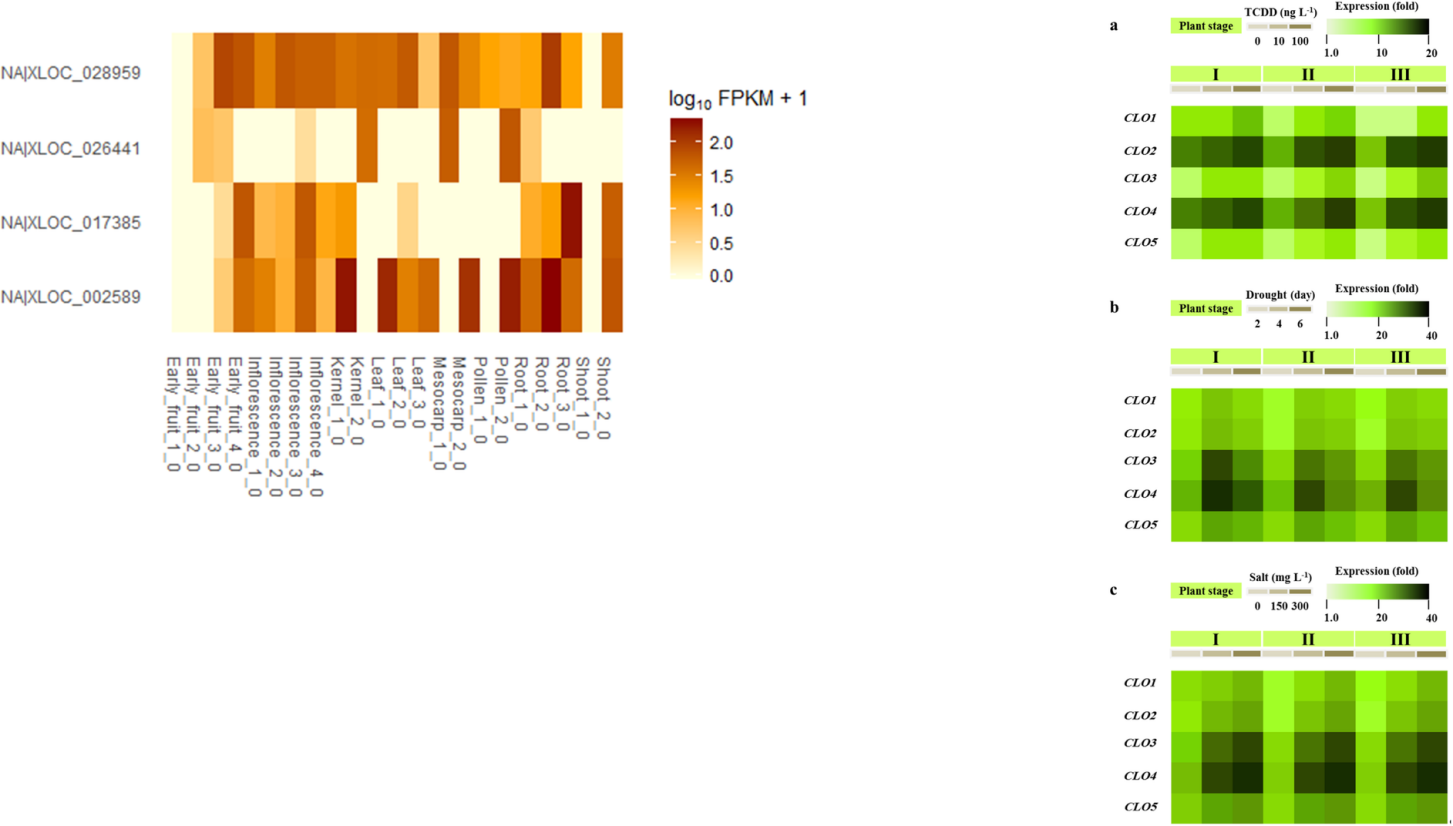
Transcriptome analysis of *CLO/PXG* gene expression in date palm and oil palm tissues. (A) The oil palm genome P5-build was used to read map the RNA seq data from Roche 454 reads (a full dataset is available from NCBI BioProject PRJNA201497). Reads from Roche/454-derived libraries were assembled into isotigs, which were blasted onto Arabidopsis thaliana gene models with a threshold of E-value < 10−5. The best-hit *A*. *thaliana* gene model was assigned to the homologue of the query isotig. To estimate expression levels of genes in mesocarp and kernel tissues, Illumina HiSeq 2000 reads from each library were mapped to assembled isotigs from all *Elaeis guineensis* reads by using the Burrows–Wheeler Aligner. Gene group expression levels were calculated as the number of mapped reads on each isotig divided by the total number of isotigs, multiplied by 100,000, and scaled by the number of genes in each gene group. Both copy number and read coverage were the mean of measures from two biological replicates. Data were analysed as described above for Roche/454 data, except that expression levels were calculated as transcripts per million tags. Identification of expression caleosin in oil palm was done using open source Tuxedo suite software[50]. (B) Transcriptional analysis of *CLO/PXG* gene expression in date palm tissues and treatments as follows: a) exposure to 0, 10 and 100 ng.L^-1^ of the dioxin, TCDD; b) drought for 2, 4 and 6 days; c) exposure to 0, 150 and 300 ng.L^-1^ NaCl. Seedlings with a radicle length of 0.5 or 2 or 4.5 cm were referred as stage I, II and III respectively.

The oil palm genome contains six *CLO/PXG*-like sequences and a heatmap analysis of their expression patterns in 22 different tissue libraries is shown in Fig 4A. Transcripts of *EgCLO2* were particularly highly expressed in developing fruit mesocarp and kernel (seed) tissues where there is considerable accumulation of storage lipids. In contrast *EgCLO1* was more highly expressed in shoot and floral tissues, while *EgCLO3* showed high but relatively even levels of expression in all tissues in a manner similar to that of a constitutive gene profile. The remaining three genes, *EgCLO4*, *5*, *6*, all responded to the same probe so these data represent the sum of their expression patterns. In comparison with the other genes, these three had much lower expression in most conditions although they showed significant upregulation in later stages of kernel, mesocarp and pollen development. These three tissues are all actively accumulating storage lipids at the stages where *EgCLO4*, *5*, *6* are upregulated which is consistent with a role in LD formation as found in other CLO/PXG proteins in other plant and algal species. The overall differentially regulated patterns of *CLO/PXG*-like gene expression in oil palm supports the conclusion that the encoded proteins carry out a range of roles in various tissues throughout the plant and that these roles occur at different developmental stages.

The date palm genome contains five CLO/PXG-like sequences but only three of these genes, *PdCLO2*, *3* and *4*, were expressed (Fig 4B) at moderate to high levels following the various treatments while *PdCLO1* and *5* were expressed at much lower levels in all cases. Exposure to the hydrophobic organic toxin, dioxin, led to a 20-fold upregulation of *PdCLO2* and *3*, while *PdCLO3* and *4* were transiently upregulated by >30-fold following drought stress and were also >20-fold upregulated following increasing exposure to NaCl. Expression of *PdCLO4* gene was higher in the young emerging shoot (plumule) than in radicle while *PdCLO2* was more expressed in radicle tissues than in plumule and very small levels of transcripts for both genes were found in petioles [74]. This expression pattern, with a strong upregulation in response to various abiotic stresses, is similar to that on other plants. It is possible that the less expressed genes *PdCLO1* and *5* have different roles, such as in abiotic stress or seed development, that were not tested in this transcriptome panel.

### Evolution of the CLO/PXG gene family in the Viridiplantae

**Fig 5:**
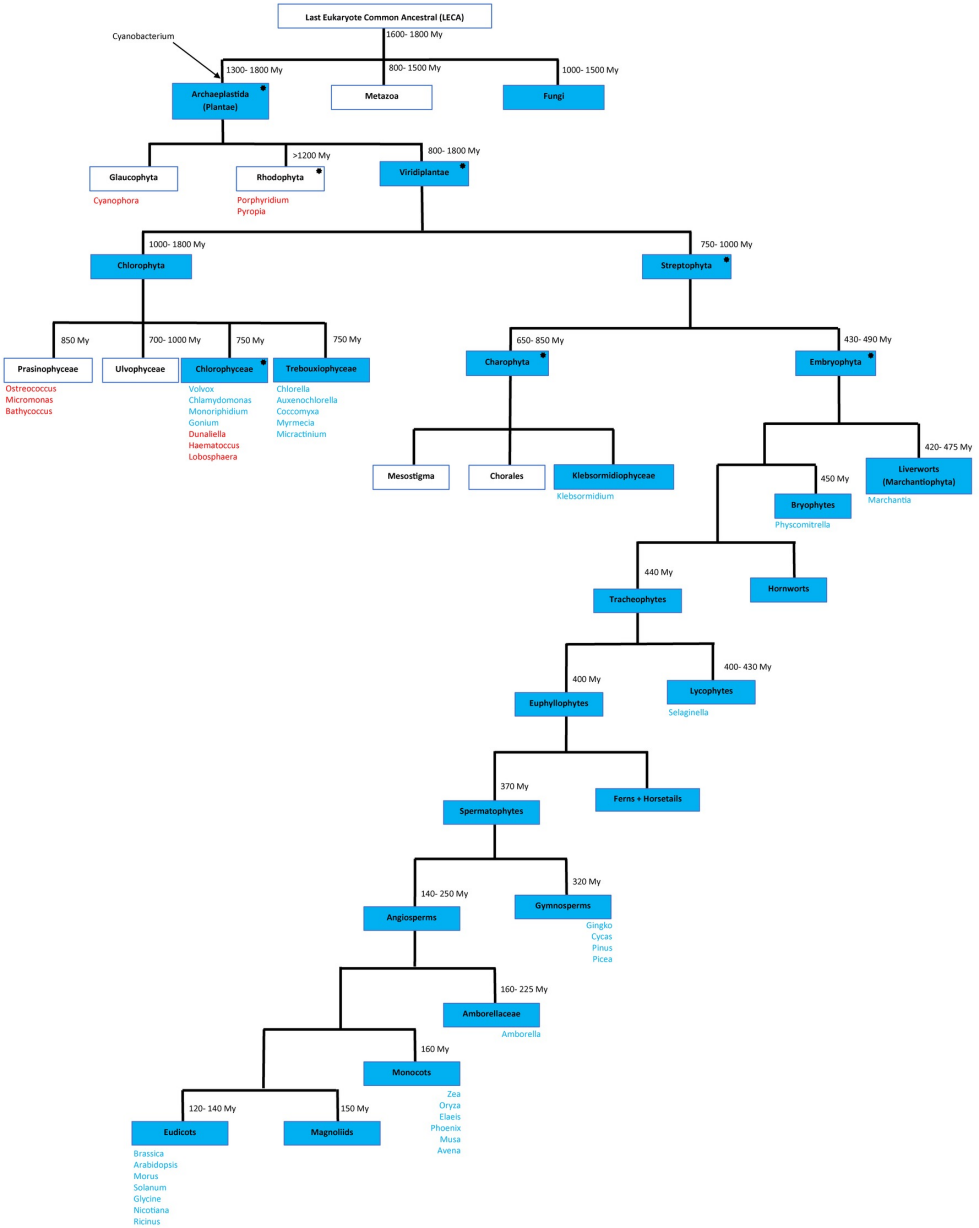
Presence of *CLO/PXG* sequences across the Viridiplantae and their estimated evolutionary divergence times. The major taxa that contain *CLO/PXG* sequences are shown as blue-shaded boxes. Individual species with one or more *CLO/PXG* sequences are shown in blue while other species where *CLO/PXG* sequences are definitely absent from their genomes are shown in brown. The estimated evolutionary divergence times of selected key taxa are shown as the number of million years ago (My). Starred major taxa are those with good evidence of monophyletic status while non-starred taxa are probably polyphyletic or paraphyletic.

The occurrence of *CLO/PXG*-like genes is depicted in the evolutionary tree shown in Fig 5. In all currently sequences Archaeplastida, *CLO/PXG*-like genes are only found in the Viridiplantae, which is one of four taxa that make up this group. The Archaeplastida (Plantae) are widely, albeit not universally, recognised as a monophyletic group of photosynthetic organisms descended from an endosymbiotic association between a heterotrophic eukaryote and a cyanobacterium that took place >2 Bya [75]. This supergroup includes all extant red and green algae plus the land plants [76]. It is now recognised that several other groups of non-Archaeplastidal photosynthetic organisms, most notably the Stramenopiles, which include diatoms and brown algae, originated separately from non-plant/algal ancestors that secondarily acquired red or green algal endosymbionts [77]. These organisms include diatoms, the genomes of which definitely lack *CLO/PXG* orthologs, although some species have recently been shown to contain a very different class of LD-binding proteins that appear to play analogous roles to CLO/PXG in LD accumulation in response to stress [78–80].

The two major groups that make up the Archaeplastida are the Viridiplantae (from Latin for ‘green plants’), and the Rhodophyta (red algae) plus a third much smaller group, the Glaucophyta [81]. These groups of photosynthetic organisms are estimated to have diverged from each other between 1–1.6 Bya [6, 82]. The Rhodophyta crown group diverged relatively early, at 1.0–1.6 Bya [75] but the earliest divergence times of the major two clades of the Viridiplantae, the Chlorophyta and the Streptophyta, both of which are generally but not universally recognised as monophyletic taxa, is less certain [6, 82]. While most estimates of divergence times of these two groups are in the range of 0.85–1.2 Bya [6, 77, 83], a more recent study suggests a later divergence at about 0.5–1.0 Bya [75]. Although molecular clocks place the origins of the Archaeplastida at between 0.9 and 1.9 Bya [75], evidence from recent molecular fossil data suggest that Cyanobacteria remained the dominant group of photosynthetic organisms until as recently as about 650 Mya, after which the eukaryotic Archaeplastida rapidly emerged as major components of aquatic, and later terrestrial, ecosystems [84].

### The CLO/PXG gene family in the Chlorophyte algae

Since *CLO/PXG*-like genes are widely distributed in both Chlorophyta and Streptophyta, the gene family probably dates from >1 Bya in a common ancestor of the entire Viridiplantae taxon. Interestingly, it is now emerging that at this stage of algal evolution many of the Viridiplantae species, including all of those that express *CLO/PXG*-like genes, began to occupy low-salt, i.e. freshwater, habitats [75]. Given the virtually universal upregulation of *CLO/PXG*-like genes in response to salt stress [14, 17, 85], it is possible that one of the primary reasons for the evolution of *CLO/PXG*-like genes is related to a move towards non-saline environments. One of the characteristic features of higher plant CLO/PXGs is the occurrence of both H- and L- isoforms [12, 40, 64, 86] and this was found in at least one of the Chlorophyte species, *Auxenochlorella protothecoides* (Fig 1B), which indicates that *CLO/PXG*-like genes had already diverged into the two isoforms at a very early stage of their evolution. Although genome sequence data are still much less complete in green algae compared with land plants, the available information enables us to trace the presence, and sometimes the absence of *CLO/PXG*-like genes in a way that allows some evolutionary inferences to be made.

The Chlorophyta are conventionally divided into four groups, namely Trebouxophyceae, Chlorophyceae, Ulvaphyceae and Prasinophyceae. There is good evidence for the Chlorophyceae being a monophyletic clade while the other three groups are highly paraphyletic[5, 83]. *CLO/PXG*-like genes are present in at least four species of Chlorophyceae (*Chlamydomonas reinhardtii*, *Gonium pectorale*, *Monoraphidium neglectum* and *Volvox carteri*) and four species of Trebouxophyceae (*Auxenochlorella protothecoides*, *Chlorella variabilis*, *C*. *vulgaris* and *Coccomyxa subellipsoidea*). Furthermore, in at least five of these algal species the biological activity of these genes is supported by transcriptome and/or biochemical studies [14, 20, 44, 87]. However, *CLO/PXG*-like genes appear to be absent from several other sequenced genomes in both the Chlorophyceae (e.g. *Dunaliella* and *Haematococcus* spp.) and Trebouxophyceae (e.g. *Lobosphaera* spp.) [88, 89]. To date no genomes of the Ulvaphyceae have been sequenced and at present only a few complete genome sequences are available from the Prasinophyceae (e.g. *Ostreococcus tauri*, *Micromonas spp*. and *Bathycoccus prasinos*), none of which contain *CLO/PXG*-like genes. However, this does not necessarily mean that the *CLO/PXG* gene family is absent from other unsequenced members of these taxa. Overall, the most parsimonious interpretation of the currently available data is that *CLO/PXG*-like genes were originally present in the ancestor of the putatively monophyletic [83] Chlorophyta clade. Similar genes are still present in several of the present day descendants of these ancestral Chlorophyta, most notably in some, but not all, Chlorophyceae and Trebouxiophyceae. However the *CLO/PXG* genes appear to have been lost in other Chlorophyta species during the >1 billion years of their subsequent evolution.

### The CLO/PXG gene family in the Streptophyte algae

The Streptophytes, including the Charophytes and Embryophytes are a monophyletic clade that diverged from the Chlorophytes >1.0 Bya [90, 91]. To date, full genomic sequence data are only available for one species of the Charophyta, namely the terrestrial and freshwater filamentous alga, *Klebsormidium nitens*, where as many as five *CLO/PXG*-like genes are also present. In addition, transcriptome data from the related species, *K*. *crenulatum* show the desiccation-induced expression of no fewer than six CLO/PXG-like genes [92]. *Klebsormidium* algae have primitive body plans and are made up of multicellular non-branching filaments that can survive on the land with substantial tolerance to novel stresses, such as drought and freezing, which do not normally occur in aquatic environments [93]. Therefore it is possible that as some Streptophyte algae became increasingly adapted to terrestrial conditions, new functions emerged for the CLO/PXG proteins inherited from their marine ancestors. Given the ubiquity of the occurrence of *CLO/PXG*-like genes in the Embryophytes (see below), it seems likely that similar genes will be found in other Charophyte species once more sequence data become available in the future.

### The CLO/PXG gene family in the Embryophytes

Embryophytes are a monophyletic group probably diverged from Charophytes about 500 Mya and there is robust fossil evidence of their presence in a variety of terrestrial environments by 430–490 Mya [4, 94–98]. The most basal groups of Embryophytes are the Liverworts and Bryophytes (mosses) each of which is represented by just one sequenced genome, namely *Marchantia polymorpha* and *Physcomitrella patens*. In both cases their genomes contain only L isoform *CLO/PXG*-like sequences, suggesting that the H isoform has been lost in these relatively primitive non-vascular, multicellular plants. However, both L and H *CLO/PXG* isoforms are present in lycophyte, *Selaginella moellendorfii*, which is a fern-like seedless vascular plant that can form true roots and has the kind of ABA signalling pathways for which the *A*. *thaliana* CLO/PXG isoform, ATCLO4, acts as a negative regulator [98, 99]. Although no true fern genomes have been sequenced, such data are available for four species of Gymnosperm, which are seed-bearing vascular, but non-flowering, higher plants. In all cases, only H isoform *CLO/PXG* are present in these genomes (Fig 1B). Therefore, in the case of this admittedly small sample of non-flowering land plants, most species contain only one of these two *CLO/PXG* isoforms.

To date *CLO/PXG-*like genes have been found in all of the >300 angiosperm species for which sequence data are available. The split between Angiosperms and other vascular plants occurred >200 Mya [100] and the former now make up the majority of the terrestrial flora in terms of biomass and species diversity[101, 102]. In all of these species our analysis suggests that there is at least one copy each of the L and H isoforms of CLO/PXG and that in many cases these genes have become highly duplicated to form relatively large families of *CLO/PXG-*like genes, many of which are differentially expressed in various developmental and/or environmental conditions. We and others have previously shown that in a range of higher plants including Arabidopsis, maize, and rice, some *CLO* genes are induced by a variety of biotic and abiotic stresses (e.g. *RD20*, *AtPXG1*, *AtPXG2*, *EFA27*, *OsCLO-2*, *OsCLO-6*, *ZmCLO7*) while other *CLO* genes are unresponsive to such stresses but may be highly expressed in lipid-storing tissues such as seeds [8, 28, 69, 70]. It is also possible to divide higher plant CLO/PXGs into two functional groups, one of which has very low epoxygenase activity when expressed under standard conditions and tends to be seed specific, while the other has high epoxygenase activity and is expressed throughout the plant[28]. In many well-characterised higher plant genomes there are large numbers of *CLO/PXG-*like genes, such as six in Arabidopsis, 11 in the diploid Brassicas, nine in rice and 12 in maize. These sequences include highly conserved regions also found in algae, plus more variable regions some of which may have arisen after the monocot/dicot divergence of about 150–160 Mya [13]. In some cases the annotated *CLO/PXG* sequences appear to be pseudogenes as they are not expressed and in some cases lack crucial CLO domains and may also contain additional non-CLO domains. An example of the latter is the presence of bZIP and PKinase domains in the non-expressed putative maize sequences, *ZmCLO1* and *ZmCLO2a* [13]and in an Arabidopsis *CLO* sequence [103].

The expansion and increasing complexity of *CLO/PXG-*like gene families in land plants is consistent with the evolution of a wider range of functions by the various CLO/PXG protein isoforms. Some of the functions relating to nutrient stress responses and LD packaging are also seen even in unicellular green algae and are probably universal for CLO/PXGs [43, 87, 104]. However, other functions such as oxylipin-based signalling [105, 106] are unique to multicellular organisms and probably evolved later, while other peroxygenase activities are related to the production of extracellular waxes such as cutin [107–109] and desiccation tolerance [35, 92, 110] so these functions would not have been required until plants became terrestrial. It is also becoming evident that CLO/PXGs have central roles in plant-pathogen responses, especially with fungi where the proteins may be involved in both host and pathogen crosstalk via oxylipin pathways [8, 9, 15, 98, 111]. In summary, the evolution of the *CLO/PXG* gene family in the Viridiplantae involves over one billion years of gradually expanding functions as these organisms increased in size and complexity and had to adapt to new forms of biotic and abiotic stress as they colonised new habitats and faced new challenges both in the sea and on land.

### Putative *CLO/PXG* genes/proteins in a bacterium and a basal opisthokont

**Fig 6:**
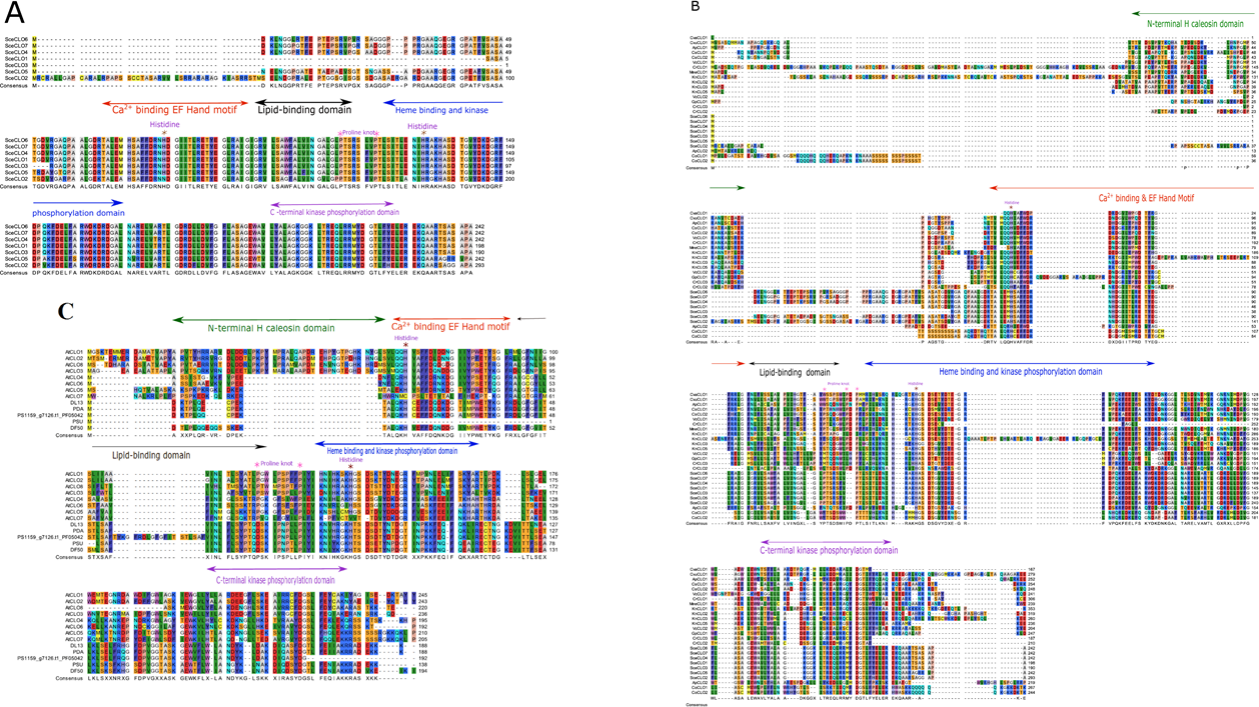
Alignments of anomalous CLO/PXG sequences from two non-Viridiplantae species. (A) Alignments of seven CLO/PXG sequences from *S*. *cellulosum*. (B) Alignments of two CLO/PXG sequences from *C*. *owczarzaki* with a range of green algal sequences. **(**C) Alignments of five putative CLO/PXG sequences from the Metazoan nematode genus, *Panagrolaimus spp*, with sequences from *A*. *thaliana*.

We found that public databases contain several DNA sequences with weak to moderate similarity to *CLO/PXG* in other major taxa apart from Viridiplantae or Fungi. In the majority of cases these can be dismissed as being potential functional members of the CLO/PXG family because the derived proteins lack canonical domains required for biological functions, such as calcium-, haem- or lipid-binding motifs. However, there are several database entries that do contain the major *CLO/PXG* motifs and these may be non-orthologous versions, i.e. xenologs, of the well-established Viridiplantae and Fungal sequences [112]. In particular, there are several annotated putative *CLO/PXG*-like sequences in the genomes of the myxobacterium, *Sorangium cellulosum*, and the basal opisthokont, *Capsaspora owczarzaki*. As shown in Fig 6A, 6B and 6C, the derived protein sequences from these two organisms contain a calcium-binding EF hand motif plus the two invariant histidine residues involved in heme binding and a lipid-binding domain, all of which are in the same locations in the protein as in the well established plant and fungal sequences. There are additional smaller regions of sequence similarity, including in putative kinase domains, which may indicate that these proteins may be regulated by phosphorylation and the predicted proteins are also of similar length to most verified CLO/PXGs. One notable absence in the *S*. *cellulosum* and *C*. *owczarzaki* sequences is the H-domain, a motif that is found close to the N terminus of many, but not all, algal and land plant CLO/PXGs (see Fig 6B). This may indicate that if these sequences were acquired via horizontal gene transfer (HGT) then they originated as plant-derived L-caleosins.

Interestingly, no *CLO/PXG*-like sequences were found in the genomes of any of the close relatives of either *S*. *cellulosum*, *C*. *owczarzaki* or *Panagrolaimus spp* for which data are available. This may indicate that the genes were originally acquired as isolated instances of HGT and then became duplicated and were retained as multiple-copy gene families due to their usefulness for the particular lifestyles of these organisms. For example, *S*. *cellulosum*

Soce56 is a highly unusual aerobic myxobacterium that has the largest bacterial genome sequenced to date at 14.8 Mb [113]. The genome of this bacterium has undergone massive expansion due to gene endo-duplication and HGT, and its lifestyle involves high levels of social behaviour and production of a wide range of secondary metabolites with complex regulatory networks including many kinases that facilitate responses to rapidly fluctuating environments [113]. With their known roles in stress responses, signal transduction, and oxylipin metabolism it can be seen why the presence of as many as seven *CLO/PXG* genes might be adaptive for *S*. *cellulosum*.

In the case of the nematode genus, *Panagrolaimus spp*, a BLAST search with the five putative *CLO/PXG* sequences gave both higher plants and fungal *CLO/PXG*s in the top few hits. Therefore, if the original gene was acquired via HGT, as seems likely it is not clear whether the donor was a plant or a fungus. One interesting feature of the putative *CLO/PXG* genes from the nematodes is that they were only found in parthenogenetic species in the genus and are functionally linked to cryptobiosis and especially to desiccation tolerance [114]. As discussed above, the algal caleosins are highly upregulated following salt stress and may also have played a role in the transition of more complex plants from aquatic to terrestrial environments with the concomitant requirement for improved tolerance to desiccation.

A third well-supported putative *CLO/PXG* gene family is present in another unusual organism, namely the eukaryote, *C*. *owczarzaki*, which is a filose amoeboid symbiont of the pulmonate snail, *Biomphalaria glabrata*. Genomic sequence data suggest that *C*. *owczarzaki* is a member of an opisthokont lineage, the Holozoa, which is more closely related to the Metazoa than to the other major multicellular opisthokont group, the Fungi [115]. Interestingly, during specific phases of its life cycle, *C*. *owczarzaki* cells accumulate and then extrude LDs, although it is not known whether these extracellular LDs contain CLO/PXGs as is found in several algae (see above) [116, 117].

A less likely candidate CLO/PXG protein has been reported in the dinoflagellate, *Symbiodinium* [43] In this case an LD-associated 20kDa protein in *Symbiodinium* cells cross-reacted with anti-CLO/PXG antibodies generated against purified sesame and cycad proteins, which could possibly imply the presence of a CLO/PXG protein in this species. However, *Symbiodinium*, which is a photosynthetic endosymbiont within the reef building coral, *Euphyllia glabrescens* is a dinoflagellate that is unrelated to either the Viridiplantae or Fungi, having acquired its plastids from red algae, which do not have *CLO/PXG* genes (see above). Therefore, in the absence of definitive sequence data for the 20kDa LD protein or its gene, it cannot be assumed to be a *bona fide* CLO/PXG.

Finally, we found a few isolated database entries of *CLO/PXG*-like sequences in genomes of taxa from which these genes are thought to be absent, most notably the Metazoa. For example, we found a *CLO/PXG*-like sequence with very strong identity to higher plants in the genome entry for the Gulf Coast tick, *Amblyomma maculatum* (GenBank: AEO32378.1). These ticks are parasites of small mammals and birds. The entry was from a shotgun transcriptome assembly that had involved the collection of several hundred million sequences from ticks kept in culture. Correspondence with the lab concerned established that the ticks used in the study also harboured some algal growths on their external surfaces and that this may have been the source of the *CLO/PXG*-like sequence. It can be concluded that this particular *CLO/PXG*-like database entry is highly likely to be erroneous and due to contamination from plant or algal sequences rather than to HGT. The take-home messages from the two examples of *Symbiodinium* and *A*. *maculatum*, are that a) considerable caution should be exercised in the use of antibody cross reactivity in the absence of sequence data and b) anomalous sequence entries in databases should be carefully scrutinised before definite conclusions are drawn.

## Conclusions

The distribution of CLO/PXG-like genes is consistent with their origin >1 billion years ago in at least two of the earliest diverging groups of the Viridiplantae, namely Chlorophyta and Streptophyta but the Viridiplantae from other Archaeplastidal groups such as the Rhodophyta and Glaucophyta. The algal CLO/PXGs have roles in lipid packaging and stress responses, especially related to dehydration and salinity. In contrast, the Embryophyte CLO/PXG proteins have a much wider spectrum of physiological roles including oxylipin signaling pathways, and may have been instrumental in the colonisation of terrestrial habitats and the subsequent diversification as the major land flora.

## Supporting information

S1 Fig Workflow of the bioinformatics analysis. The workflow details the procedures performed during data analysis.
(PDF)

S2 Fig Transmembrane domain prediction. Data from 34 species are displayed showing predicted TM domains.
(PDF)

S3 Fig Secondary structure prediction. Data from 34 species are displayed showing predicted secondary structures.
(PDF)

S4 Fig Intron/exon analysis. Summary chart of overall intron/exon numbers in each of the 67 analysed genes.
(TIFF)

S5 Fig Predicted intron-exon sequence blocks across selected *CLO/PXGs* from 34 species. Due to unavailability of full genome sequence data for some genes in public databases, intron-exons were analysed for 60 out of 67 sequences from 34 species. Black blocks indicate exons and grey blocks indicate introns. Each sequence is scaled based on its length as indicated immediately on the left side just above the block.
(PNG)

S1 Table PCR primers used in this study. (PDF)

S2 Table List of the 131 representative CLO/PXG proteins from 34 species used in this study. The species marked in green represents 67 CLO/PXG sequences from 34 species used in this study.
(XLSX)

S3 Table List of 34 species with their tax ID and no of caleosin genes per species. (XLSX)

S4 Table Transmembrane (TM) domain prediction. The table indicates the location of each TM domain by starting and end point. The length of each TM is consistent at about 21 residues.
(XLSX)

S5 Table Intron-exon list. Predicted intron/exon locations for all 67 analysed sequences. Blocks represent exons and light lines show introns.
(XLSX)

## References

[1] Shen Y, Xie J, Liu R-d, Ni X-f, Wang X-h, Li Z-x, et al Genomic analysis and expression investigation of caleosin gene family in Arabidopsis. 2014;448(4):365–71. doi: 10.1016/j.bbrc.2014.04.115 24796675 10.1016/j.bbrc.2014.04.115

[2] Song W, Qin Y, Zhu Y, Yin G, Wu N, Li Y, et al Delineation of plant caleosin residues critical for functional divergence, positive selection and coevolution. BMC Evol Biol. 2014;14:124 Epub 2014/06/09. doi: 10.1186/1471-2148-14-124 ; PubMed Central PMCID: PMCPMC4057654. 24913827 10.1186/1471-2148-14-124 PMC4057654

[3] Hanano A, Almousally I, Shaban M, Rahman F, Blee E, murphy Dj. Biochemical, transcriptional and bioinformatic analysis of lipid droplets from seeds of date palm (Phoenix dactyliferaL.) and their use as potent sequestration agents against the toxic pollutant, 2,3,7,8-tetrachlorinated dibenzo-p-dioxin. Frontiers in Plant Science. 2016;7. 10.3389/fpls.2016.00836 PMC4896926 27375673

[4] Magallón S, Hilu KW, Quandt D. Land plant evolutionary timeline: gene effects are secondary to fossil constraints in relaxed clock estimation of age and substitution rates. American Journal of Botany 100, 556–573. 2013;100:556–73. 10.3732/ajb.1200416 23445823

[5] Moczydlowska M, Landing E, Zang W, Palacios T. Proterozoic phytoplankton and timing of Chlorophyte algae origins. 2011;54:721–33.

[6] Ruhfel BR, Gitzendanner MA, Soltis PS, Soltis DE, Burleigh JG. From algae to angiosperms-inferring the phylogeny of green plants (Viridiplantae) from 360 plastid genomes. BMC Evol Biol. 2014;14:23 Epub 2014/02/17. doi: 10.1186/1471-2148-14-23 ; PubMed Central PMCID: PMCPMC3933183. 24533922 10.1186/1471-2148-14-23 PMC3933183

[7] Næsted H, Frandsen GI, Jauh GY, Hernandez-Pinzon I, Nielsen HB, Murphy DJ, et al Caleosins: Ca2+-binding proteins associated with lipid bodies. Plant Mol Biol. 2000;44 doi: 10.1023/a:1026564411918 10.1023/a:1026564411918 11197322

[8] Partridge M, Murphy DJ. Roles of a membrane-bound caleosin and putative peroxygenase in biotic and abiotic stress responses in Arabidopsis. Plant Physiol Biochem. 2009;47(9):796–806. Epub 2009/05/09. doi: 10.1016/j.plaphy.2009.04.005 . 19467604 10.1016/j.plaphy.2009.04.005

[9] Hanano A, Almousally I, Shaban M, Blee E. A Caleosin-Like Protein with Peroxygenase Activity Mediates Aspergillus flavus Development, Aflatoxin Accumulation, and Seed Infection. Appl Environ Microbiol. 2015;81(18):6129–44. Epub 2015/06/26. doi: 10.1128/AEM.00867-15 ; PubMed Central PMCID: PMCPMC4542254. 26116672 10.1128/AEM.00867-15 PMC4542254

[10] Hanano A, Burcklen M, Flenet M, Ivancich A, Louwagie M, Garin J, et al Plant seed peroxygenase is an original heme-oxygenase with an EF-hand calcium binding motif. J Biol Chem. 2006;281 doi: 10.1074/jbc.M605395200 16956885 10.1074/jbc.M605395200

[11] Purkrtova Z, Le Bon C, Kralova B, Ropers M-H, Anton M, Chardot T. Caleosin of Arabidopsis thaliana: Effect of Calcium on Functional and Structural Properties. Journal of Agricultural and Food Chemistry. 2008;56(23):11217–24. doi: 10.1021/jf802305b 19012406 10.1021/jf802305b

[12] Khalil HB, Brunetti SC, Pham UM, Maret D, Laroche A, Gulick PJ. Characterization of the caleosin gene family in the Triticeae. BMC Genomics. 2014;15:239 Epub 2014/03/27. doi: 10.1186/1471-2164-15-239 ; PubMed Central PMCID: PMCPMC3986672. 24673767 10.1186/1471-2164-15-239 PMC3986672

[13] Lizong H, Jinjie G, Yongfeng Z, Liying Z, Yaqun H, Jingtang C. Molecular characterization and evolutionary analysis of the putative caleosin gene family in maize (Zea mays). 2014;16:261–8.

[14] Charuchinda P, Waditee-Sirisattha R, Kageyama H, Yamada D, Sirisattha S, Tanaka Y, et al Caleosin from Chlorella vulgaris TISTR 8580 is salt-induced and heme-containing protein. Bioscience Biotechnology and Biochemistry. 2015;79(7):1119–24. doi: 10.1080/09168451.2015.1010480 PubMed PMID: WOS:000357330200014. 25703935 10.1080/09168451.2015.1010480

[15] Hanano A, Bessoule JJ, Heitz T, Blée E. Involvement of the caleosin/peroxygenase RD20 in the control of cell death during Arabidopsis responses to pathogens. Plant Signal Behav. 2015;10(4):e991574 doi: 10.4161/15592324.2014.991574 ; PubMed Central PMCID: PMCPMC4622875. 25830533 10.4161/15592324.2014.991574 PMC4622875

[16] Purkrtová Z, Chardot T, Froissard M. N-terminus of seed caleosins is essential for lipid droplet sorting but not for lipid accumulation. 2015;579(Supplement C):47–54. 10.1016/j.abb.2015.05.008 26032334

[17] Chen JC, Tsai CC, Tzen JT. Cloning and secondary structure analysis of caleosin, a unique calcium-binding protein in oil bodies of plant seeds. Plant Cell Physiol. 1999;40(10):1079–86. . 10589521 10.1093/oxfordjournals.pcp.a029490

[18] Poxleitner M, Rogers SW, Samuels AL, Browse J, Rogers JC. A role for caleosin in degradation of oil-body storage lipids during seed germination. Plant J. 2006;47 doi: 10.1111/j.1365-313X.2006.02845.x 16961733 10.1111/j.1365-313X.2006.02845.x

[19] Jiang PL, Chen JC, Chiu ST, Tzen JT. Stable oil bodies sheltered by a unique caleosin in cycad megagametophytes. Plant Physiol Biochem. 2009;47(11–12):1009–16. Epub 2009/07/15. doi: 10.1016/j.plaphy.2009.07.004 . 19635673 10.1016/j.plaphy.2009.07.004

[20] Lin IP, Jiang PL, Chen CS, Tzen JT. A unique caleosin serving as the major integral protein in oil bodies isolated from Chlorella sp. cells cultured with limited nitrogen. Plant Physiol Biochem. 2012;61:80–7. Epub 2012/09/26. doi: 10.1016/j.plaphy.2012.09.008 . 23085585 10.1016/j.plaphy.2012.09.008

[21] Hernandez-Pinzon I, Patel K, Murphy DJ. The Brassica napus calcium-binding protein, caleosin, has distinct endoplasmic reticulum- and lipid body-associated isoforms. 2001;39(7):615–22.

[22] Murphy DJ, Hernández-Pinzón I, Patel K. Roles of lipid bodies and lipid-body proteins in seeds and other tissues. J Plant Physiol. 2001;158:471–8.

[23] Murphy DJ. Biogenesis and functions of lipid bodies in animals, plants and microorganisms. Prog Lipid Res. 2001;40 doi: 10.1016/s0163-7827(01)00013-3 10.1016/s0163-7827(01)00013-3 11470496

[24] Murphy DJ. The dynamic roles of intracellular lipid droplets: from archaea to mammals. Protoplasma. 2012;249(3):541–85. Epub 2011/10/15. doi: 10.1007/s00709-011-0329-7 . 22002710 10.1007/s00709-011-0329-7

[25] Blee E, Flenet M, Boachon B, Fauconnier ML. A non-canonical caleosin from Arabidopsis efficiently epoxidizes physiological unsaturated fatty acids with complete stereoselectivity. Febs Journal. 2012;279(20):3981–95. doi: 10.1111/j.1742-4658.2012.08757.x PubMed PMID: WOS:000309447400017. 22913587 10.1111/j.1742-4658.2012.08757.x

[26] Meesapyodsuk D, Qiu X. A peroxygenase pathway involved in the biosynthesis of epoxy fatty acids in oat. Plant Physiol. 2011;157(1):454–63. Epub 2011/07/22. doi: 10.1104/pp.111.178822 ; PubMed Central PMCID: PMCPMC3165891. 21784965 10.1104/pp.111.178822 PMC3165891

[27] Benaragama I. Molecular analysis of fatty acid peroxygenase involved in the biosynthesis of epoxy fatty acids in oats (Avena sativa) [MSc]. Available online at: https://ecommons.usask.ca/handle/10388/ETD-2015-10-22752015.

[28] Benaragama I, Meesapyodsuk D, Beattie AD, Qiu X. Identification and functional analysis of new peroxygenases in oat. Planta. 2017 Epub 2017/06/29. doi: 10.1007/s00425-017-2729-1 . 28664421 10.1007/s00425-017-2729-1

[29] Hamberg M, Hamberg G. Peroxygenase-Catalyzed Fatty Acid Epoxidation in Cereal Seeds (Sequential Oxidation of Linoleic Acid into 9(S),12(S),13(S)-Trihydroxy-10(E)-Octadecenoic Acid). Plant Physiol. 1996;110(3):807–15. ; PubMed Central PMCID: PMCPMC157780. 12226220 10.1104/pp.110.3.807 PMC157780

[30] Hanano A, Burcklen M, Flenet M, Ivancich A, Louwagie M, Garin J, et al Plant seed peroxygenase is an original heme-oxygenase with an EF-hand calcium binding motif. J Biol Chem. 2006;281(44):33140–51. Epub 2006/09/06. doi: 10.1074/jbc.M605395200 . 16956885 10.1074/jbc.M605395200

[31] Fuchs C, Schwab W. Epoxidation, hydroxylation and aromatization is catalyzed by a peroxygenase from Solanum lycopersicum. 2013;96(Supplement C):52–60.

[32] Pasaribu B, Chen CS, Liao YK, Jiang PL, Tzen JT. Identification of caleosin and oleosin in oil bodies of pine pollen. Plant Physiol Biochem. 2017;111:20–9. Epub 2016/11/18. doi: 10.1016/j.plaphy.2016.11.010 . 27889638 10.1016/j.plaphy.2016.11.010

[33] Sham A, Al-Azzawi A, Al-Ameri S, Al-Mahmoud B, Awwad F, Al-Rawashdeh A, et al Transcriptome analysis reveals genes commonly induced by Botrytis cinerea infection, cold, drought and oxidative stresses in Arabidopsis. PLoS One. 2014;9(11):e113718 Epub 2014/11/25. doi: 10.1371/journal.pone.0113718 ; PubMed Central PMCID: PMCPMC4244146. 25422934 10.1371/journal.pone.0113718 PMC4244146

[34] Frandsen G, Müller-Uri F, Nielsen M, Mundy J, Skriver K. Novel plant Ca2+-binding protein expressed in response to abscisic acid and osmotic stress. J Biol Chem. 1996;271 doi: 10.1074/jbc.271.1.343 10.1074/jbc.271.1.343 8550584

[35] Liu MS, Chien CT, Lin TP. Constitutive components and induced gene expression are involved in the desiccation tolerance of Selaginella tamariscina. Plant Cell Physiol. 2008;49(4):653–63. Epub 2008/03/07. doi: 10.1093/pcp/pcn040 . 18326542 10.1093/pcp/pcn040

[36] Aubert Y, Vile D, Pervent M, Aldon D, Ranty B, Simonneau T, et al RD20, a stress-inducible caleosin, participates in stomatal control, transpiration and drought tolerance in Arabidopsis thaliana. Plant Cell Physiol. 2010;51 doi: 10.1093/pcp/pcq155 20952421 10.1093/pcp/pcq155

[37] Neves-Borges AC, Guimarães-Dias F, Cruz F, Mesquita RO, Nepomuceno AL, Romano E, et al Expression pattern of drought stress marker genes in soybean roots under two water deficit systems. Genet Mol Biol. 2012;35(1 (suppl)):212–21. doi: 10.1590/S1415-47572012000200003 ; PubMed Central PMCID: PMCPMC3392874. 22802707 10.1590/S1415-47572012000200003 PMC3392874

[38] Sham A, Moustafa K, Al-Ameri S, Al-Azzawi A, Iratni R, AbuQamar S. Identification of Arabidopsis candidate genes in response to biotic and abiotic stresses using comparative microarrays. PLoS One. 2015;10(5):e0125666 Epub 2015/05/01. doi: 10.1371/journal.pone.0125666 ; PubMed Central PMCID: PMCPMC4416716. 25933420 10.1371/journal.pone.0125666 PMC4416716

[39] Hanano A, Almousally I, Shaban M, Rahman F, Blee E, Murphy DJ. Biochemical, Transcriptional, and Bioinformatic Analysis of Lipid Droplets from Seeds of Date Palm (Phoenix dactylifera L.) and Their Use as Potent Sequestration Agents against the Toxic Pollutant, 2,3,7,8-Tetrachlorinated Dibenzo-p-Dioxin. Front Plant Sci. 2016;7:836 Epub 2016/06/08. doi: 10.3389/fpls.2016.00836 ; PubMed Central PMCID: PMCPMC4896926. 27375673 10.3389/fpls.2016.00836 PMC4896926

[40] Khalil HB, Wang Z, Wright JA, Ralevski A, Donayo AO, Gulick PJ. Heterotrimeric Gα subunit from wheat (Triticum aestivum), GA3, interacts with the calcium-binding protein, Clo3, and the phosphoinositide-specific phospholipase C, PI-PLC1. Plant Molecular Biology. 2011;77(1):145 doi: 10.1007/s11103-011-9801-1 21725861 10.1007/s11103-011-9801-1

[41] Pasaribu B, Lin IP, Chen CS, Lu CY, Jiang PL. Nutrient limitation in Auxenochlorella protothecoides induces qualitative changes of fatty acid and expression of caleosin as a membrane protein associated with oil bodies. Biotechnol Lett. 2014;36(1):175–80. doi: 10.1007/s10529-013-1332-1 . 24078127 10.1007/s10529-013-1332-1

[42] Blanc G, Agarkova I, Grimwood J, Kuo A, Brueggeman A, Dunigan DD, et al The genome of the polar eukaryotic microalga Coccomyxa subellipsoidea reveals traits of cold adaptation. Genome Biol. 2012;13(5):R39 Epub 2012/05/25. doi: 10.1186/gb-2012-13-5-r39 ; PubMed Central PMCID: PMCPMC3446292. 22630137 10.1186/gb-2012-13-5-r39 PMC3446292

[43] Pasaribu B, Chung TY, Chen CS, Wang SL, Jiang PL, Tzen JT. Identification of caleosin and two oleosin isoforms in oil bodies of pine megagametophytes. Plant Physiol Biochem. 2014;82:142–50. Epub 2014/06/05. doi: 10.1016/j.plaphy.2014.05.015 . 24954070 10.1016/j.plaphy.2014.05.015

[44] Hemschemeier A, Casero D, Liu B, Benning C, Pellegrini M, Happe T, et al Copper response regulator1-dependent and -independent responses of the Chlamydomonas reinhardtii transcriptome to dark anoxia. Plant Cell. 2013;25(9):3186–211. Epub 2013/09/06. doi: 10.1105/tpc.113.115741 ; PubMed Central PMCID: PMCPMC3809527. 24014546 10.1105/tpc.113.115741 PMC3809527

[45] Jiang PL, Jauh GY, Wang CS, Tzen JT. A unique caleosin in oil bodies of lily pollen. Plant Cell Physiol. 2008;49(9):1390–5. Epub 2008/07/16. doi: 10.1093/pcp/pcn103 . 18632804 10.1093/pcp/pcn103

[46] Froissard M, D'andréa S, Boulard C, Chardot T. Heterologous expression of AtClo1, a plant oil body protein, induces lipid accumulation in yeast. FEMS Yeast Res. 2009;9(3):428–38. Epub 2009/02/10. doi: 10.1111/j.1567-1364.2009.00483.x . 19220478 10.1111/j.1567-1364.2009.00483.x

[47] Zienkiewicz K, Castro AJ, Alché JD, Zienkiewicz A, Suárez C, Rodríguez-García MI. Identification and localization of a caleosin in olive (Olea europaea L.) pollen during in vitro germination. J Exp Bot. 2010;61 doi: 10.1093/jxb/erq022 20164143 10.1093/jxb/erq022 PMC2837266

[48] Zienkiewicz K, Zienkiewicz A, Rodríguez-García MI, Castro AJ. Characterization of a caleosin expressed during olive (Olea europaeaL.) pollen ontogeny. BMC Plant Biology. 2011;11(1):122 doi: 10.1186/1471-2229-11-122 21884593 10.1186/1471-2229-11-122 PMC3180362

[49] Hu R, Fan C, Li H, Zhang Q, Fu YF. Evaluation of putative reference genes for gene expression normalization in soybean by quantitative real-time RT-PCR. BMC Molecular Biology. 2009;10:93 Epub 2009/09/30. doi: 10.1186/1471-2199-10-93 ; PubMed Central PMCID: PMC2761916. 19785741 10.1186/1471-2199-10-93 PMC2761916

[50] Singh R, Ong-Abdullah M, Low ET, Manaf MA, Rosli R, Nookiah R, et al Oil palm genome sequence reveals divergence of interfertile species in Old and New worlds. Nature. 2013;500(7462):335–9. Epub 2013/07/24. doi: 10.1038/nature12309 ; PubMed Central PMCID: PMCPMC3929164. 23883927 10.1038/nature12309 PMC3929164

[51] Trapnell C, Roberts A, Goff L, Pertea G, Kim D, Kelley DR, et al Differential gene and transcript expression analysis of RNA-seq experiments with TopHat and Cufflinks. Nat Protoc. 2012;7(3):562–78. Epub 2012/03/01. doi: 10.1038/nprot.2012.016 ; PubMed Central PMCID: PMCPMC3334321. 22383036 10.1038/nprot.2012.016 PMC3334321

[52] Camacho C, Coulouris G, Avagyan V, Ma N, Papadopoulos J, Bealer K, et al BLAST+: architecture and applications. BMC Bioinformatics. 2009;10(1):421 doi: 10.1186/1471-2105-10-421 20003500 10.1186/1471-2105-10-421 PMC2803857

[53] Kearse M, Moir R, Wilson A, Stones-Havas S, Cheung M, Sturrock S, et al Geneious Basic: an integrated and extendable desktop software platform for the organization and analysis of sequence data. Bioinformatics. 2012;28(12):1647–9. Epub 2012/04/27. doi: 10.1093/bioinformatics/bts199 ; PubMed Central PMCID: PMCPMC3371832. 22543367 10.1093/bioinformatics/bts199 PMC3371832

[54] Finn RD, Attwood TK, Babbitt PC, Bateman A, Bork P, Bridge AJ, et al InterPro in 2017-beyond protein family and domain annotations. Nucleic Acids Res. 2017;45(D1):D190–D9. Epub 2016/11/29. doi: 10.1093/nar/gkw1107 ; PubMed Central PMCID: PMCPMC5210578. 27899635 10.1093/nar/gkw1107 PMC5210578

[55] Gasteiger E, Gattiker A, Hoogland C, Ivanyi I, Appel RD, Bairoch A. ExPASy: The proteomics server for in-depth protein knowledge and analysis. Nucleic Acids Res. 2003;31(13):3784–8. ; PubMed Central PMCID: PMCPMC168970. 12824418 10.1093/nar/gkg563 PMC168970

[56] Artimo P, Jonnalagedda M, Arnold K, Baratin D, Csardi G, de Castro E, et al ExPASy: SIB bioinformatics resource portal. Nucleic Acids Res. 2012;40(Web Server issue):W597–603. Epub 2012/05/31. doi: 10.1093/nar/gks400 ; PubMed Central PMCID: PMCPMC3394269. 22661580 10.1093/nar/gks400 PMC3394269

[57] Bailey TL, Boden M, Buske FA, Frith M, Grant CE, Clementi L, et al MEME SUITE: tools for motif discovery and searching. Nucleic Acids Res. 2009;37(Web Server issue):W202–8. Epub 2009/05/20. doi: 10.1093/nar/gkp335 ; PubMed Central PMCID: PMCPMC2703892. 19458158 10.1093/nar/gkp335 PMC2703892

[58] Bailey TL, Johnson J, Grant CE, Noble WS. The MEME Suite. Nucleic Acids Res. 2015;43(W1):W39–49. Epub 2015/05/07. doi: 10.1093/nar/gkv416 ; PubMed Central PMCID: PMCPMC4489269. 25953851 10.1093/nar/gkv416 PMC4489269

[59] Keller O, Odronitz F, Stanke M, Kollmar M, Waack S. Scipio: Using protein sequences to determine the precise exon/intron structures of genes and their orthologs in closely related species. BMC Bioinformatics. 2008;9(1):278 doi: 10.1186/1471-2105-9-278 18554390 10.1186/1471-2105-9-278 PMC2442105

[60] Larkin MA, Blackshields G, Brown NP, Chenna R, McGettigan PA, McWilliam H, et al Clustal W and Clustal X version 2.0. Bioinformatics. 2007;23(21):2947–8. doi: 10.1093/bioinformatics/btm404 17846036 10.1093/bioinformatics/btm404

[61] Sievers F, Wilm A, Dineen D, Gibson TJ, Karplus K, Li W, et al Fast, scalable generation of high-quality protein multiple sequence alignments using Clustal Omega. Mol Syst Biol. 2011;7:539 Epub 2011/10/11. doi: 10.1038/msb.2011.75 ; PubMed Central PMCID: PMCPMC3261699. 21988835 10.1038/msb.2011.75 PMC3261699

[62] Sayle RA, Milner-White EJ. RASMOL: biomolecular graphics for all. 1995;20(9):374–6. 10.1016/s0968-0004(00)89080-5 7482707

[63] Rambaut A. FigTree. Available at: http://tree.bio.ed.ac.uk. 2009.

[64] Hu L, Li S, Gao W. Expression, divergence and evolution of the caleosin gene family in Brassica rapa. 2013;65(3):863–76.

[65] Shen Y, Jia Q, Liu M, Li Z, Wang L, Zhao C, et al Genome-wide characterization and phylogenetic and expression analyses of the caleosin gene family in soybean, common bean and barrel medic. 2016b;68:48–66.

[66] Shen Y, Liu M, Wang L, Li Z, Taylor D, Li Z, et al Identification, duplication, evolution and expression analyses of caleosins in Brassica plants and Arabidopsis subspecies. Molecular Genetics and Genomics. 2016a;291:971–88. doi: 10.1007/s00438-015-1156-x 26786939 10.1007/s00438-015-1156-x

[67] Odronitz F, Pillmann H, Keller O, Waack S, Kollmar M. WebScipio: an online tool for the determination of gene structures using protein sequences. BMC Genomics. 2008;9:422 Epub 2008/09/18. doi: 10.1186/1471-2164-9-422 ; PubMed Central PMCID: PMCPMC2644328. 18801164 10.1186/1471-2164-9-422 PMC2644328

[68] Hatje K, Keller O, Hammesfahr B, Pillmann H, Waack S, Kollmar M. Cross-species protein sequence and gene structure prediction with fine-tuned Webscipio 2.0 and Scipio. BMC Res Notes. 2011;4:265 Epub 2011/07/28. doi: 10.1186/1756-0500-4-265 ; PubMed Central PMCID: PMCPMC3162530. 21798037 10.1186/1756-0500-4-265 PMC3162530

[69] Aubert Y, Vile D, Pervent M, Aldon D, Ranty B, Simonneau T, et al RD20, a stress-inducible caleosin, participates in stomatal control, transpiration and drought tolerance in Arabidopsis thaliana. Plant Cell Physiol. 2010;51(12):1975–87. Epub 2010/10/14. doi: 10.1093/pcp/pcq155 . 20952421 10.1093/pcp/pcq155

[70] Yu WL, Ansari W, Schoepp NG, Hannon MJ, Mayfield SP, Burkart MD. Modifications of the metabolic pathways of lipid and triacylglycerol production in microalgae. Microb Cell Fact. 2011;10:91 Epub 2011/11/02. doi: 10.1186/1475-2859-10-91 ; PubMed Central PMCID: PMCPMC3234195. 22047615 10.1186/1475-2859-10-91 PMC3234195

[71] Umate P. Comparative genomics of the lipid-body-membrane proteins oleosin, caleosin and steroleosin in magnoliophyte, lycophyte and bryophyte. Genomics Proteomics Bioinformatics. 2012;10(6):345–53. Epub 2012/12/07. doi: 10.1016/j.gpb.2012.08.006 ; PubMed Central PMCID: PMCPMC5054715. 23317702 10.1016/j.gpb.2012.08.006 PMC5054715

[72] Chang Y, Wang S, Sekimoto S, Aerts AL, Choi C, Clum A, et al Phylogenomic Analyses Indicate that Early Fungi Evolved Digesting Cell Walls of Algal Ancestors of Land Plants. Genome Biol Evol. 2015;7(6):1590–601. Epub 2015/05/14. doi: 10.1093/gbe/evv090 ; PubMed Central PMCID: PMCPMC4494064. 25977457 10.1093/gbe/evv090 PMC4494064

[73] Berbee ML, James TY, Strullu-Derrien C. Early Diverging Fungi: Diversity and Impact at the Dawn of Terrestrial Life. Annual Review of Microbiology. 2017;71(1):41–60. 10.1146/annurev-micro-030117-020324 28525299

[74] Hanano A, Almousally I, Shaban M, Rahman F, Hassan M, Murphy DJ. Specific Caleosin/Peroxygenase and Lipoxygenase Activities Are Tissue-Differentially Expressed in Date Palm (Phoenix dactylifera L.) Seedlings and Are Further Induced Following Exposure to the Toxin 2,3,7,8-tetrachlorodibenzo-p-dioxin. Front plant sci. 2016;7:2025 Epub 2017/01/24. doi: 10.3389/fpls.2016.02025 ; PubMed Central PMCID: PMC5216026. 28111588 10.3389/fpls.2016.02025 PMC5216026

[75] Sánchez-Baracaldo P, Raven JA, Pisani D, Knoll AH. Early photosynthetic eukaryotes inhabited low-salinity habitats. Proc Natl Acad Sci U S A. 2017;114(37):E7737–E45. Epub 2017/08/14. doi: 10.1073/pnas.1620089114 ; PubMed Central PMCID: PMCPMC5603991. 28808007 10.1073/pnas.1620089114 PMC5603991

[76] Mackiewicz P, Gagat P. Monophyly of Archaeplastida supergroup and relationships among its lineages in the light of phylogenetic and phylogenomic studies. Are we close to a consensus? 2014;83:263–80.

[77] Derelle R, López-García P, Timpano H, Moreira D. A Phylogenomic Framework to Study the Diversity and Evolution of Stramenopiles = Heterokonts). Mol Biol Evol. 2016;33(11):2890–8. Epub 2016/08/10. doi: 10.1093/molbev/msw168 ; PubMed Central PMCID: PMCPMC5482393. 27512113 10.1093/molbev/msw168 PMC5482393

[78] Shemesh Z, Leu S, Khozin-Goldberg I, Didi-Cohen S, Zarka A, Boussiba S. Inducible expression of Haematococcus oil globule protein in the diatom Phaeodactylum tricornutum: Association with lipid droplets and enhancement of TAG accumulation under nitrogen starvation. 2016;18(Supplement C):321–31.

[79] Yoneda K, Yoshida M, Suzuki I, Watanabe MM. Identification of a Major Lipid Droplet Protein in a Marine Diatom Phaeodactylum tricornutum. Plant Cell Physiol. 2016;57(2):397–406. Epub 2016/01/06. doi: 10.1093/pcp/pcv204 . 26738549 10.1093/pcp/pcv204

[80] Maeda Y, Nojima D, Yoshino T, Tanaka T. Structure and properties of oil bodies in diatoms. Philos Trans R Soc Lond B Biol Sci. 2017;372(1728). doi: 10.1098/rstb.2016.0408 ; PubMed Central PMCID: PMCPMC5516117. 28717018 10.1098/rstb.2016.0408 PMC5516117

[81] Qiu H, Yoon HS, Bhattacharya D. Red Algal Phylogenomics Provides a Robust Framework for Inferring Evolution of Key Metabolic Pathways. PLoS Currents. 2016;8:ecurrents.tol.7b037376e6d84a1be34af756a4d90846. 10.1371/currents.tol.7b037376e6d84a1be34af756a4d90846 PMC5164836 28018750

[82] Chan CX, Yang EC, Banerjee T, Yoon HS, Martone PT, Estevez JM, et al Red and green algal monophyly and extensive gene sharing found in a rich repertoire of red algal genes. Curr Biol. 2011;21(4):328–33. doi: 10.1016/j.cub.2011.01.037 . 21315598 10.1016/j.cub.2011.01.037

[83] Leliaert F, Smith DR, Moreau H, Herron MD, Verbruggen H, Delwiche CF, et al Phylogeny and Molecular Evolution of the Green Algae. Critical Reviews in Plant Sciences. 2012;31(1):1–46.

[84] Brocks JJ, Jarrett AJM, Sirantoine E, Hallmann C, Hoshino Y, Liyanage T. The rise of algae in Cryogenian oceans and the emergence of animals. 2017;548(7669):578–81. doi: 10.1038/nature23457 28813409 10.1038/nature23457

[85] Partridge M, Murphy D. Roles of a membrane-bound caleosin and putative peroxygenase in biotic and abiotic stress responses in Arabidopsis. Plant Physiol Biochem. 2009;47 doi: 10.1016/j.plaphy.2009.04.005 19467604 10.1016/j.plaphy.2009.04.005

[86] Hyun TK, Kumar D, Cho YY, Hyun HN, Kim JS. Computational identification and phylogenetic analysis of the oil-body structural proteins, oleosin and caleosin, in castor bean and flax. Gene. 2013;515(2):454–60. Epub 2012/12/08. doi: 10.1016/j.gene.2012.11.065 . 23232356 10.1016/j.gene.2012.11.065

[87] Ouyang LL, Chen SH, Li Y, Zhou ZG. Transcriptome analysis reveals unique C4-like photosynthesis and oil body formation in an arachidonic acid-rich microalga Myrmecia incisa Reisigl H4301. BMC Genomics. 2013;14:396 Epub 2013/06/13. doi: 10.1186/1471-2164-14-396 ; PubMed Central PMCID: PMCPMC3686703. 23759028 10.1186/1471-2164-14-396 PMC3686703

[88] Davidi L, Katz A, Pick U. Characterization of major lipid droplet proteins from Dunaliella. Planta. 2012;236(1):19–33. Epub 2012/01/10. doi: 10.1007/s00425-011-1585-7 . 22231009 10.1007/s00425-011-1585-7

[89] Yao L, Tan KWM, Tan TW, Lee YK. Exploring the transcriptome of non-model oleaginous microalga Dunaliella tertiolecta through high-throughput sequencing and high performance computing. BMC Bioinformatics. 2017;18(1):122 doi: 10.1186/s12859-017-1551-x 28228091 10.1186/s12859-017-1551-x PMC5322580

[90] Delwiche C. The Genomes of Charophyte Algae. Advances in Botanical Research. 2016;78:255–70.

[91] De Vries J, M Archibald J. Plant evolution: landmarks on the path to terrestrial life. New Phytologist. 2017;217:1428–34. 10.1111/nph.14975 29318635

[92] Holzinger A, Kaplan F, Blaas K, Zechmann B, Komsic-Buchmann K, Becker B. Transcriptomics of desiccation tolerance in the streptophyte green alga Klebsormidium reveal a land plant-like defense reaction. PLoS One. 2014;9(10):e110630 Epub 2014/10/23. doi: 10.1371/journal.pone.0110630 ; PubMed Central PMCID: PMCPMC4207709. 25340847 10.1371/journal.pone.0110630 PMC4207709

[93] Hori K, Maruyama F, Fujisawa T, Togashi T, Yamamoto N, Seo M, et al Klebsormidium flaccidum genome reveals primary factors for plant terrestrial adaptation. Nat Commun. 2014;5:3978 Epub 2014/05/28. doi: 10.1038/ncomms4978 ; PubMed Central PMCID: PMCPMC4052687. 24865297 10.1038/ncomms4978 PMC4052687

[94] Heckman DS, Geiser DM, Eidell BR, Stauffer RL, Kardos NL, Hedges SB. Molecular evidence for the early colonization of land by fungi and plants. Science. 2001;293(5532):1129–33. doi: 10.1126/science.1061457 . 11498589 10.1126/science.1061457

[95] Sanderson MJ, Thorne JL, Wikström N, Bremer K. Molecular evidence on plant divergence times. Am J Bot. 2004;91(10):1656–65. doi: 10.3732/ajb.91.10.1656 . 21652315 10.3732/ajb.91.10.1656

[96] Steemans P, Hérissé AL, Melvin J, Miller MA, Paris F, Verniers J, et al Origin and radiation of the earliest vascular land plants. Science. 2009;324(5925):353 doi: 10.1126/science.1169659 . 19372423 10.1126/science.1169659

[97] Wellman C, Strother P. The terrestrial biota prior to the origin of land plants (embryophytes): a review of the evidence. Palaeontology. 2015;58:601–27.

[98] Zhu X, Dunand C, Snedden W, Galaud JP. CaM and CML emergence in the green lineage. Trends Plant Sci. 2015;20(8):483–9. Epub 2015/06/23. doi: 10.1016/j.tplants.2015.05.010 . 26115779 10.1016/j.tplants.2015.05.010

[99] Kim YY, Jung KW, Yoo KS, Jeung JU, Shin JS. A stress-responsive caleosin-like protein, AtCLO4, acts as a negative regulator of ABA responses in Arabidopsis. Plant Cell Physiol. 2011;52(5):874–84. Epub 2011/04/06. doi: 10.1093/pcp/pcr039 . 21471120 10.1093/pcp/pcr039

[100] Morris JL, Puttick MN, Clark JW, Edwards D, Kenrick P, Pressel S, et al The timescale of early land plant evolution. Proc Natl Acad Sci U S A. 2018;115(10):E2274–E83. Epub 2018/02/20. doi: 10.1073/pnas.1719588115 ; PubMed Central PMCID: PMCPMC5877938. 29463716 10.1073/pnas.1719588115 PMC5877938

[101] Bell CD, Soltis DE, Soltis PS. The age and diversification of the angiosperms re-revisited. Am J Bot. 2010;97(8):1296–303. Epub 2010/07/19. doi: 10.3732/ajb.0900346 . 21616882 10.3732/ajb.0900346

[102] Zeng L, Zhang Q, Sun R, Kong H, Zhang N, Ma H. Resolution of deep angiosperm phylogeny using conserved nuclear genes and estimates of early divergence times. 2014;5:4956 doi: 10.1038/ncomms5956 25249442 10.1038/ncomms5956 PMC4200517

[103] Girke T, Todd J, Ruuska S, White J, Benning C, Ohlrogge J. Microarray analysis of developing Arabidopsis seeds. Plant Physiol. 2000;124(4):1570–81. ; PubMed Central PMCID: PMCPMC59856. 11115875 10.1104/pp.124.4.1570 PMC59856

[104] Zienkiewicz K, Du ZY, Ma W, Vollheyde K, Benning C. Stress-induced neutral lipid biosynthesis in microalgae—Molecular, cellular and physiological insights. Biochim Biophys Acta. 2016;1861(9 Pt B):1269–81. Epub 2016/02/13. doi: 10.1016/j.bbalip.2016.02.008 . 26883557 10.1016/j.bbalip.2016.02.008

[105] Feng H, Wang XM, Sun YF, Wang XJ, Chen XM, Guo J, et al Cloning and characterization of a calcium binding EF-hand protein gene TaCab1 from wheat and its expression in response to Puccinia striiformis f. sp tritici and abiotic stresses. Molecular Biology Reports. 2011;38(6):3857–66. doi: 10.1007/s11033-010-0501-8 PubMed PMID: WOS:000291656800030. 21110112 10.1007/s11033-010-0501-8

[106] Hanano A, Almousally I, Shaban M, Rahman F, Hassan M, Murphy DJ. Specific Caleosin/Peroxygenase and Lipoxygenase Activities Are Tissue-Differentially Expressed in Date Palm (Phoenix dactylifera L.) Seedlings and Are Further Induced Following Exposure to the Toxin 2,3,7,8-tetrachlorodibenzo-p-dioxin. Front Plant Sci. 2016;7:2025 Epub 2017/01/06. doi: 10.3389/fpls.2016.02025 ; PubMed Central PMCID: PMCPMC5216026. 28111588 10.3389/fpls.2016.02025 PMC5216026

[107] Blee E, Schuber F. Biosynthesis of cutin monomers: involvement of a lipoxygenase/peroxygenase pathway. Plant Journal. 1993;4:113–23.

[108] Lequeu J, Fauconnier M, Chammaï A, Bronner R, Blée E. Formation of plant cuticle: evidence for the occurrence of the peroxygenase pathway. 2003;36:155–64. 14535881 10.1046/j.1365-313x.2003.01865.x

[109] Kondo S, Hori K, Sasaki-Sekimoto Y, Kobayashi A, Kato T, Yuno-Ohta N, et al Primitive Extracellular Lipid Components on the Surface of the Charophytic Alga Klebsormidium flaccidum and Their Possible Biosynthetic Pathways as Deduced from the Genome Sequence. Front Plant Sci. 2016;7:952 Epub 2016/06/30. doi: 10.3389/fpls.2016.00952 ; PubMed Central PMCID: PMCPMC4927632. 27446179 10.3389/fpls.2016.00952 PMC4927632

[110] Villegente M, Marmey P, Job C, Galland M, Cueff G, Godin B, et al A Combination of Histological, Physiological, and Proteomic Approaches Shed Light on Seed Desiccation Tolerance of the Basal Angiosperm Amborella trichopoda. Proteomes. 2017;5(3). Epub 2017/07/28. doi: 10.3390/proteomes5030019 ; PubMed Central PMCID: PMCPMC5620536. 28788068 10.3390/proteomes5030019 PMC5620536

[111] Richerioux N. Analysis of gene expression in barley upon aphid attack, Thesis, University François Rabelais [MSc Thesis]. Available online at: www.diva-portal.org/diva/getDocument?urn_nbn_se_sh_diva-1289-1__fulltext.pdf2007.

[112] Darby CA, Stolzer M, Ropp PJ, Barker D, Durand D. Xenolog classification. Bioinformatics. 2017;33(5):640–9. doi: 10.1093/bioinformatics/btw686 . 27998934 10.1093/bioinformatics/btw686 PMC5860392

[113] Han K, Li ZF, Peng R, Zhu LP, Zhou T, Wang LG, et al Extraordinary expansion of a Sorangium cellulosum genome from an alkaline milieu. Sci Rep. 2013;3:2101 doi: 10.1038/srep02101 ; PubMed Central PMCID: PMCPMC3696898. 23812535 10.1038/srep02101 PMC3696898

[114] Schiffer PH, Danchin E, Burnell AM, Schiffer A-M, Creevey C, Wong S, et al Signatures of the evolution of parthenogenesis and cryptobiosis in the genomes of panagrolaimid nematodes. bioRxiv. 2017 doi: 10.1101/159152 10.1016/j.isci.2019.10.039 PMC6889759 31759330

[115] Suga H, Chen Z, de Mendoza A, Sebé-Pedrós A, Brown MW, Kramer E, et al The Capsaspora genome reveals a complex unicellular prehistory of animals. Nat Commun. 2013;4:2325 doi: 10.1038/ncomms3325 ; PubMed Central PMCID: PMCPMC3753549. 23942320 10.1038/ncomms3325 PMC3753549

[116] Owczarzak A, Stibbs HH, Bayne CJ. The destruction of Schistosoma mansoni mother sporocysts in vitro by amoebae isolated from Biomphalaria glabrata: an ultrastructural study. J Invertebr Pathol. 1980;35(1):26–33. . 7365267 10.1016/0022-2011(80)90079-8

[117] Stibbs HH, Owczarzak A, Bayne CJ, DeWan P. Schistosome sporocyst-killing Amoebae isolated from Biomphalaria glabrata. J Invertebr Pathol. 1979;33(2):159–70. . 501126 10.1016/0022-2011(79)90149-6

