# Supplementary figures and images for: Evolutionary and genomic analysis of the caleosin/peroxygenase (CLO/PXG) gene/protein families in the Viridiplantae
Source: PLoS One. 2018 May 17;13(5):e0196669. doi: 10.1371/journal.pone.0196669 (PMC5957377; doi:10.1371/journal.pone.0196669)

Data Collection

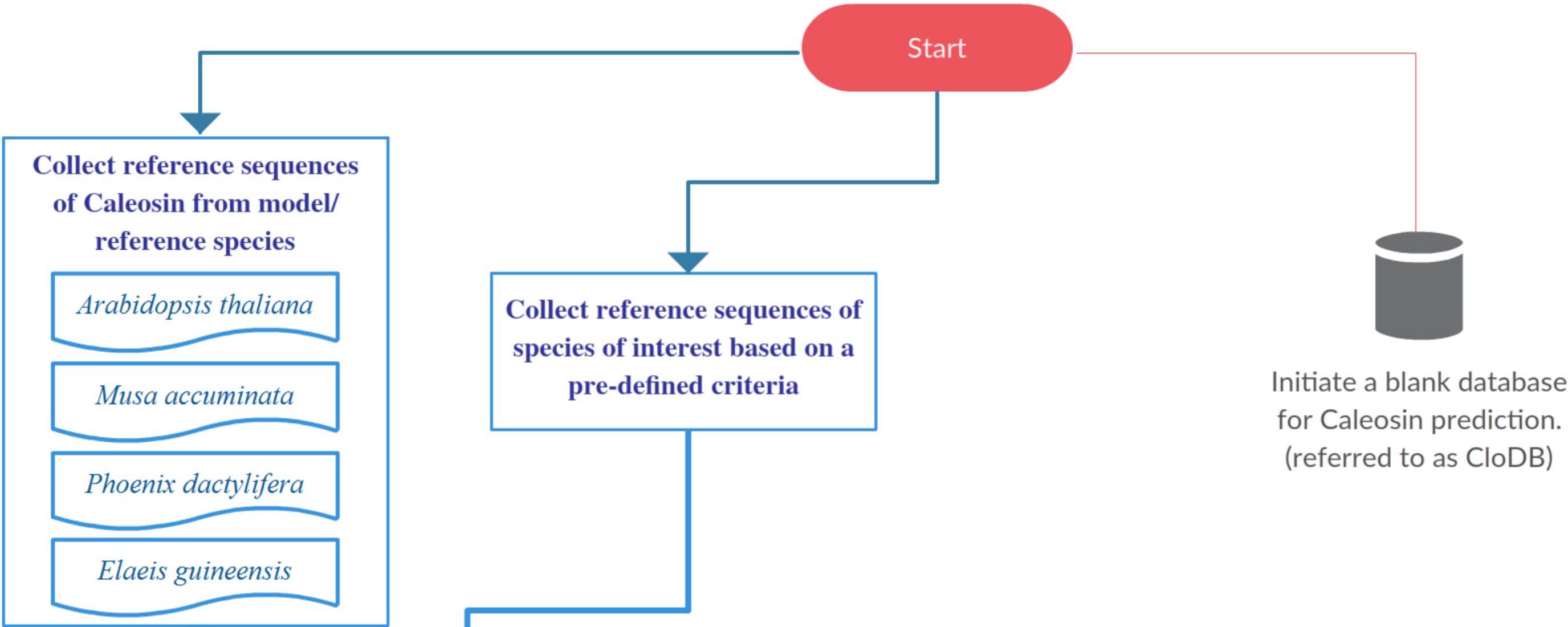

Candidate Caleosin Like Genes

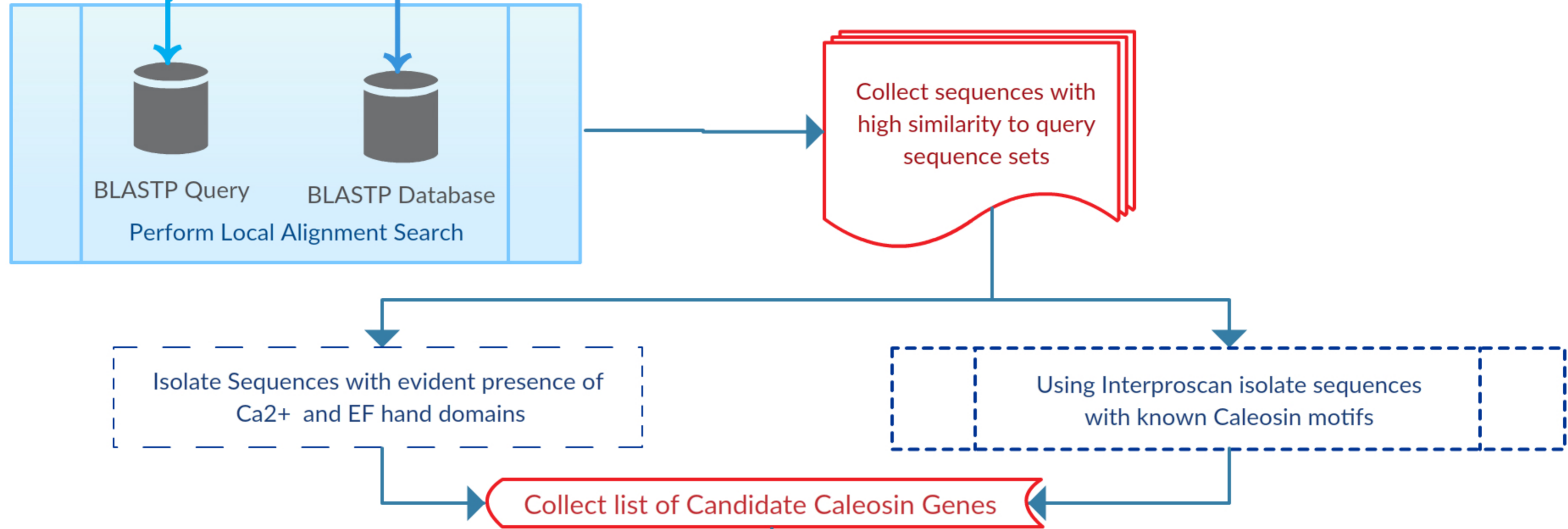

Assessment of Candidates

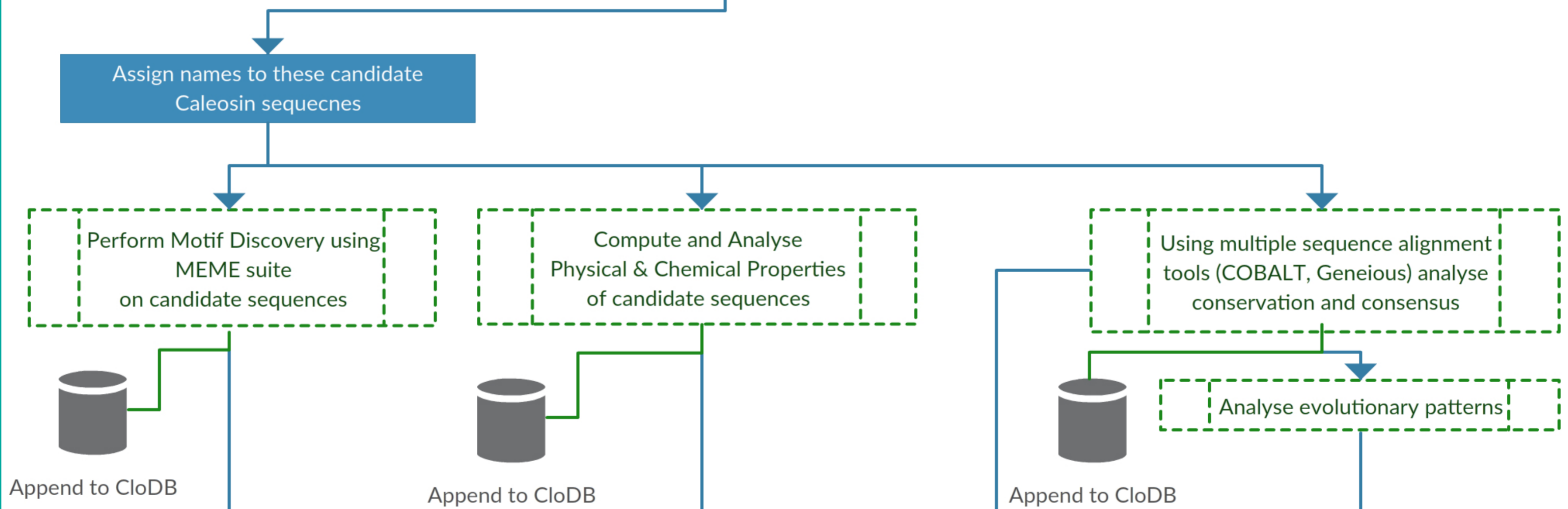

Caleosin Database

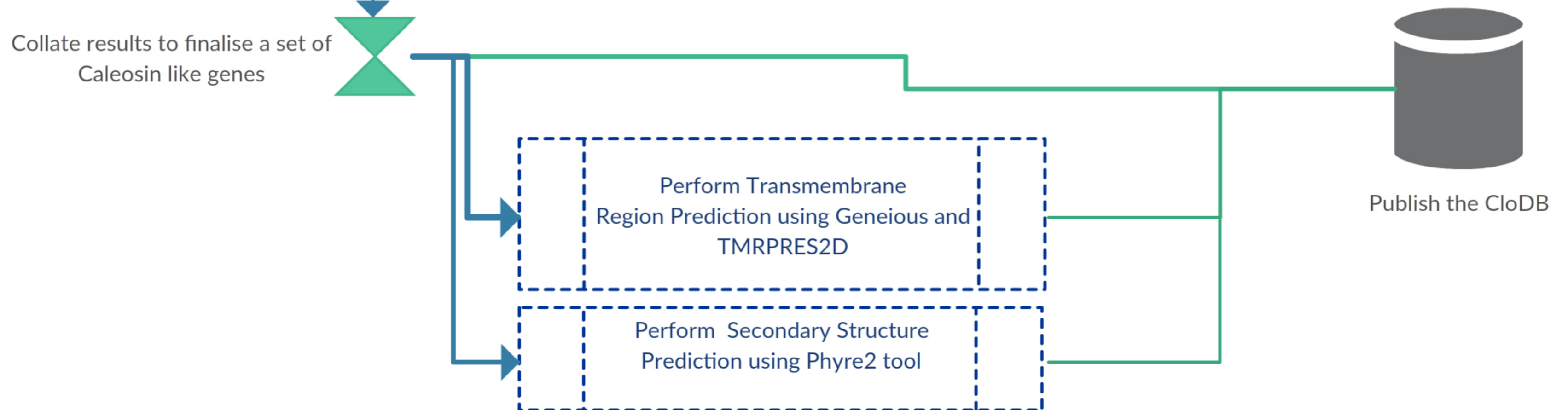

Supplement: S1 Fig — The workflow details the procedures performed during data analysis. (PDF) [file pone.0196669.s001.pdf]

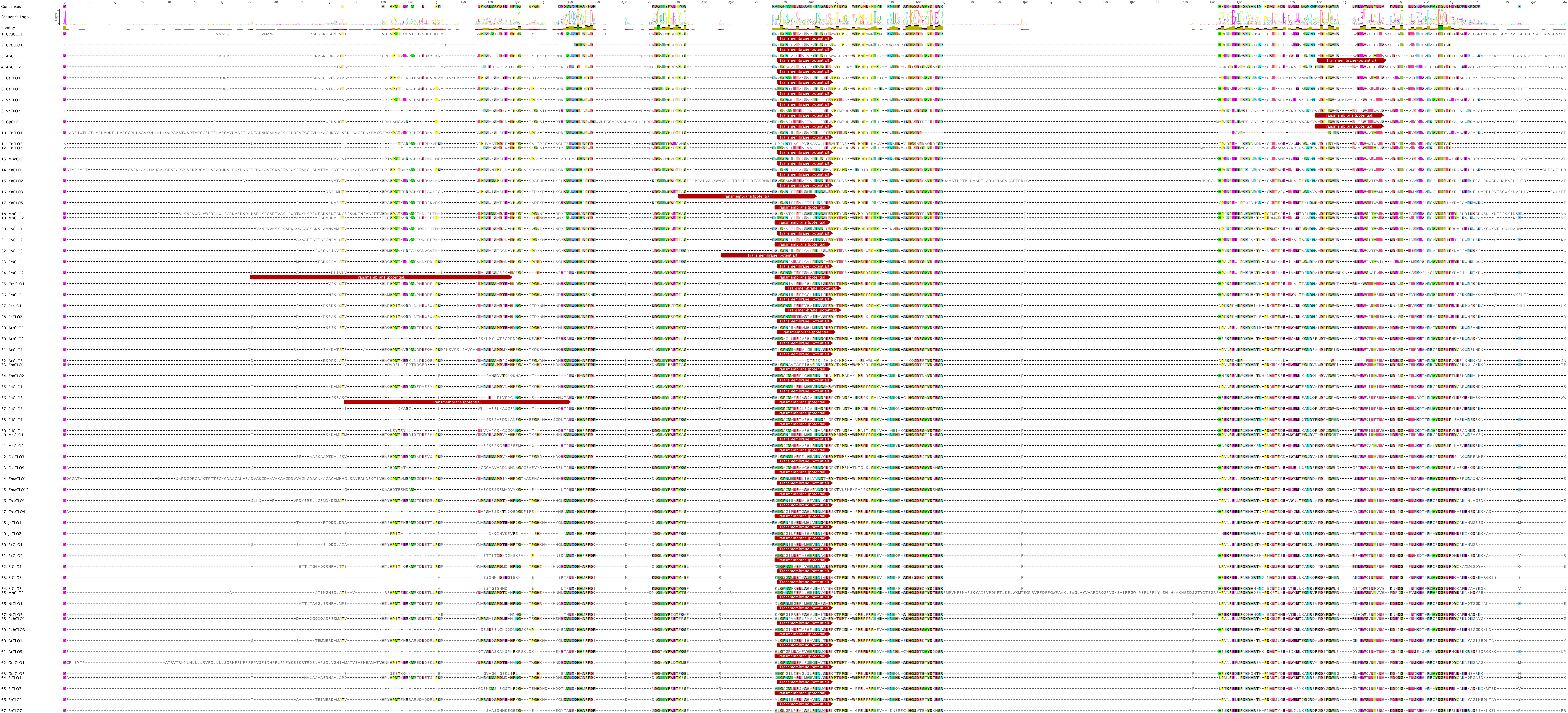

Supplement: S2 Fig — Data from 34 species are displayed showing predicted TM domains. (PDF) [file pone.0196669.s002.pdf]

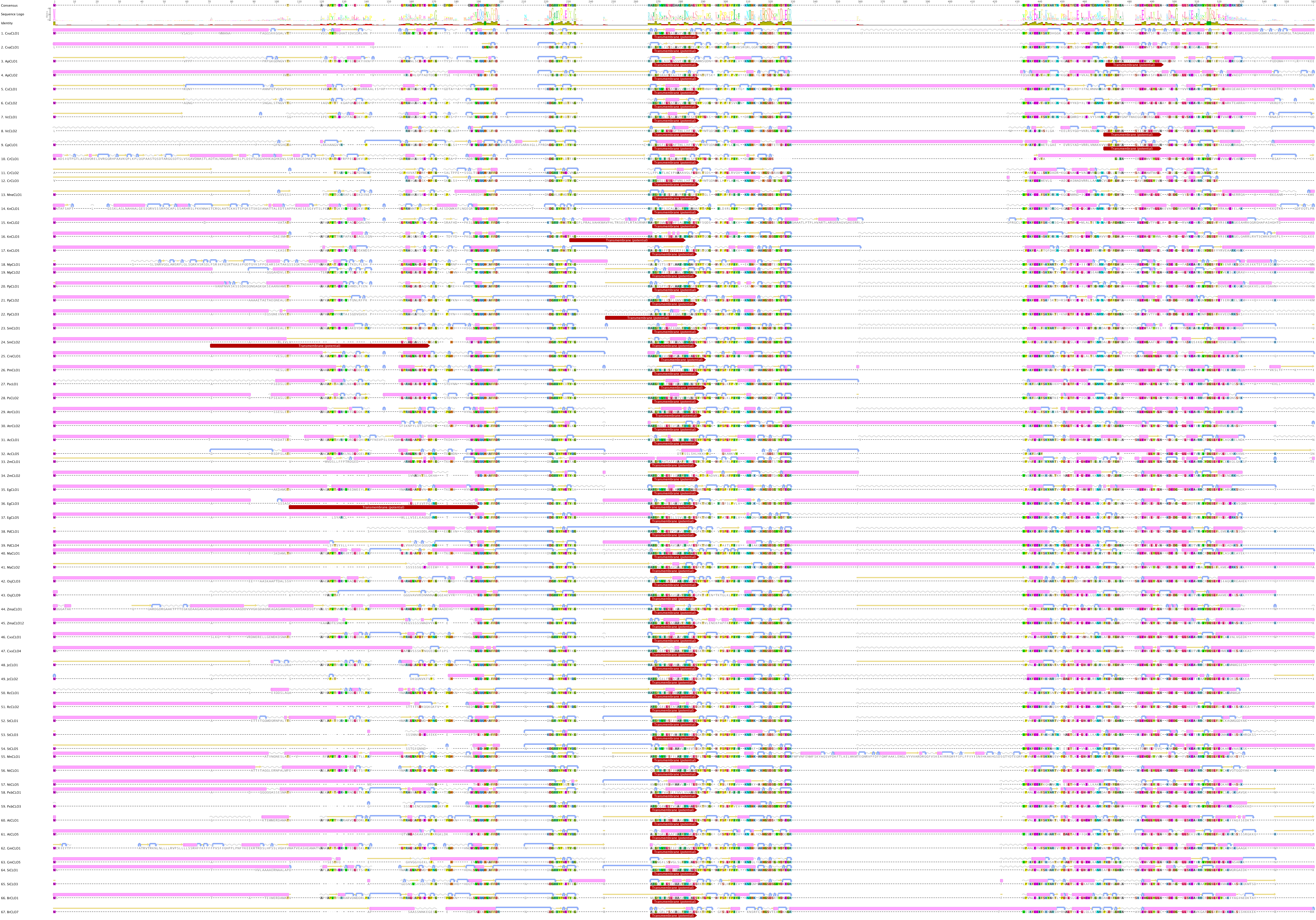

Supplement: S3 Fig — Data from 34 species are displayed showing predicted secondary structures. (PDF) [file pone.0196669.s003.pdf]

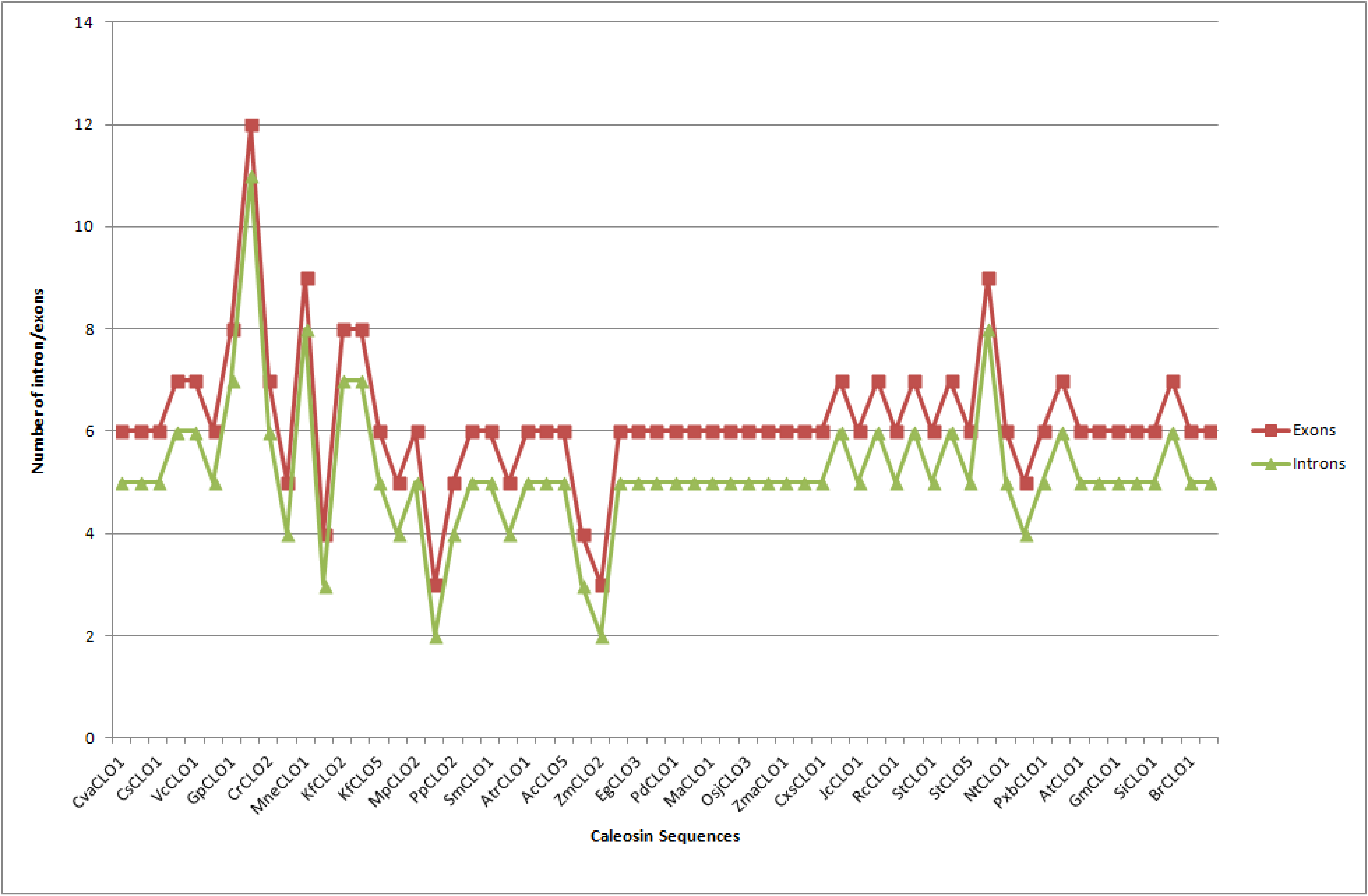

Supplement: S4 Fig — Summary chart of overall intron/exon numbers in each of the 67 analysed genes. (TIFF) [file pone.0196669.s004.tiff]

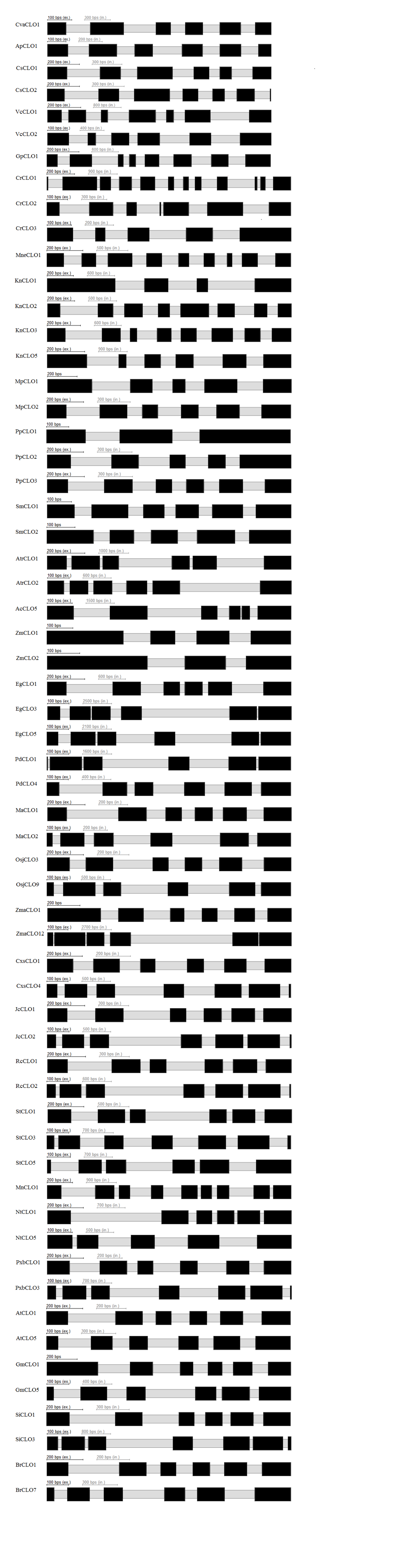

Supplement: S5 Fig — Due to unavailability of full genome sequence data for some genes in public databases, intron-exons were analysed for 60 out of 67 sequences from 34 species. Black blocks indicate exons and grey blocks indicate introns. Each sequence is scaled based on its length as indicated immediately on the left side just above the block. (PNG) [file pone.0196669.s005.png]
