# Supplementary material for: Evolutionary and genomic analysis of the caleosin/peroxygenase (CLO/PXG) gene/protein families in the Viridiplantae
Source: PLoS One. 2018 May 17;13(5):e0196669. doi: 10.1371/journal.pone.0196669 (PMC5957377; doi:10.1371/journal.pone.0196669)
Supplement: S1 Table — (PDF) [file pone.0196669.s006.pdf]

S1 Table Date palm Primers used in this study

Target gene	Order Locus	Forward/Revers Primers (5'-3')	Amplicon (bp)
CLO1_DATE	LOC103717338	GGCTACCTCCACTGCTTTCT<br>CAGTCAGGTCGCCACTGTTT	116
CLO2_DATE	LOC103696186	GGCGTCCTCATCGTTACCTT<br>GTTCCGGTCAAAGAAGGCGA	106
CLO3_DATE	LOC103696190	ACCCGACAGAGACCTACGAA<br>GATTGCCGGTAGGCTTCCAT	125
CLO4_DATE	LOC103715420	AGGCTATCAGGCGTTGCTTT<br>AGCCAAGGATTCGAGCAGTC	106
CLO5_DATE	LOC103711900	AGCGACATGCTGCCTTCTTT<br>ATCAAAACAACGCCTGGTGG	140
Actin-1	AT2G37620	CGGTATTGTGTTGGACTCTGG<br>CAGCAAGGTCAAGACGGAGT	98
Tubulin-β7	AT2G29550	GAGTGGAGTTACCTGCTGCCT<br>ATGTAGACGAGGGAACGGAA	94
